# Efficient allelic-drive in *Drosophila*

**DOI:** 10.1038/s41467-019-09694-w

**Published:** 2019-04-09

**Authors:** Annabel Guichard, Tisha Haque, Marketta Bobik, Xiang-Ru S. Xu, Carissa Klanseck, Raja Babu Singh Kushwah, Mateus Berni, Bhagyashree Kaduskar, Valentino M. Gantz, Ethan Bier

**Affiliations:** 1 Section of Cell and Developmental Biology, University of California, San Diego, 9500 Gilman Drive, La Jolla, CA 92093-0335 USA; 2 Tata Institute for Genetics and Society-India (TIGS), TIGS Center at inStem, Bangalore, 560065 India; 3 Instituto de Ciências Biomédicas (ICB), Universidade Federal do Rio de Janeiro, Av. Carlos Chagas Filho 373, Ilha do Fundão, Rio de Janeiro, 21941-902 RJ Brazil; 4 Post-graduate Program in Morphological Sciences, Federal University of Rio de Janeiro (PCM/UFRJ), Rio de Janeiro, 21941-902 RJ Brazil; 5 Tata Institute for Genetics and Society-UCSD, La Jolla, CA 92093-0335 USA

## Abstract

Gene-drive systems developed in several organisms result in super-Mendelian inheritance of transgenic insertions. Here, we generalize this “active genetic” approach to preferentially transmit allelic variants (allelic-drive) resulting from only a single or a few nucleotide alterations. We test two configurations for allelic-drive: one, copy-cutting, in which a non-preferred allele is selectively targeted for Cas9/guide RNA (gRNA) cleavage, and a more general approach, copy-grafting, that permits selective inheritance of a desired allele located in close proximity to the gRNA cut site. We also characterize a phenomenon we refer to as lethal-mosaicism that dominantly eliminates NHEJ-induced mutations and favors inheritance of functional cleavage-resistant alleles. These two efficient allelic-drive methods, enhanced by lethal mosaicism and a trans-generational drive process we refer to as “shadow-drive”, have broad practical applications in improving health and agriculture and greatly extend the active genetics toolbox.

## Abstract (web summary)

Gene-drives use CRISPR-Cas9 to be transmitted in a super-Mendelian fashion. Here the authors develop an allelic-drive for selective inheritance of a desired allele.

## Introduction

Efficient super-Mendelian inheritance of transgenic insertional elements has been demonstrated in flies^1^, mosquitoes^2,3^, yeast^4^, and mice^5^. While numerous potentially impactful applications of such so-called gene-drive systems have been proposed^6,7^, they are currently limited to copying relatively large DNA cargo sequences (~1–10 Kb). Many desired genetic traits (e.g., drought tolerance in plants, crop yield, pest-resistance, or insecticide sensitivity), however, result from allelic variants altering only one or a few base pairs. An efficient system for super-Mendelian inheritance of such subtle genetic variants would accelerate a wide array of efforts to disseminate favorable traits throughout populations or to assemble complex genotypes consisting of point-mutant alleles in combination with insertional transgenes for a multitude of research and applied purposes.

**Fig. 1:**
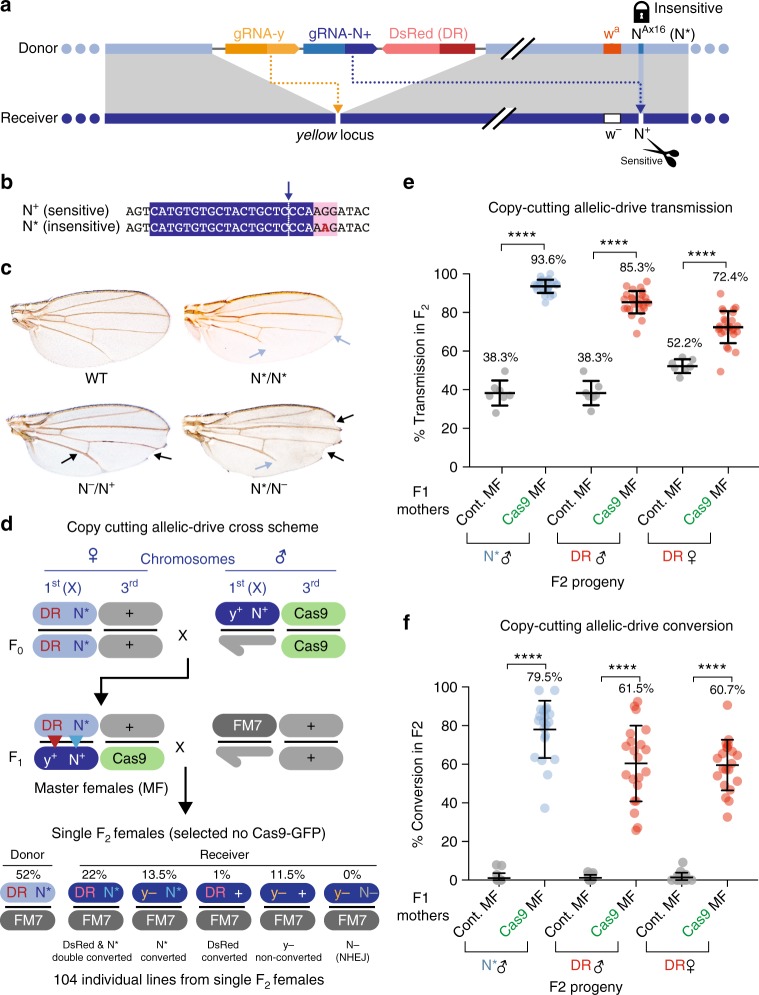
Super-Mendelian inheritance of a dominant *Notch* allele in *Drosophila*. **a** Scheme depicting a DsRed-marked *y*^<ccN>^ CopyCat element^7^ that carries two gRNAs: (1) gRNA-y (yellow), which drives copying of the *y*^<ccN>^ element at the *yellow* locus; and (2) gRNA-N+ (blue), which directs cleavage of the sensitive (S) wild-type *Notch* allele *N*^+S^ (scissors icon) to drive Super-Mendelian inheritance of the cleavage-insensitive (IS) *N*^Ax16^ allele (N*, lock icon). Cas9 is provided in trans. **b** DNA sequence of the gRNA-N+ target site on the sensitive wild-type *Notch* allele (*N*^+^), and the cleavage-insensitive *N*^Ax16^ allele (N*) is highlighted in blue, and PAM site in pink. The critical G → A substitution conferring cleavage insensitivity in *N*^Ax16^ mutants is bold in red. The Cas9/gRNA cleavage site is indicated with a dashed line. **c** Wing phenotypes of wild-type (WT), *N*^Ax16^ homozygous (N*/N*), *N*^−^ loss-of-function heterozygous (*N*^−^/*N*^+^), and *N*^Ax16^/*N*^−^ heterozygous (N^*^/*N*^−^) *Drosophila* adults. **d** Crossing scheme used to generate F1 “master females” and genotype classes of 104 isogenic lines from single F2 females (detailed analysis in Table S2). X donor chromosome carrying the DsRed marked *y*^<ccN>^ element (DR) and the *N*^Ax16^ (N*) allele appears in light blue. WT (++) cut sensitive receiver chromosome is in dark blue. Third chromosome carrying a GFP-marked transgene expressing Cas9 (*vasaCas9*) is depicted in green, and wild-type (+) chromosomes appear in light gray. The multiply inverted FM7 balancer chromosome is depicted in dark gray. Red and blue arrowheads indicate copying of the *y*^<ccN>^ element and the *N*^Ax16^ allele, respectively. **e** Percentage of transmission of *y*^<ccN>^ (DR, red circles) and *N*^Ax16^ allele (N*, blue circles) in the presence or absence (gray circles) of Cas9. *p* Value intervals for this and all subsequent unpaired parametric *t* test analysis: ****p < 0.0001; ***p < 0.001; *p < 0.05. Bars denote mean value and standard deviation in this and all subsequent graphs. **f** Percentage conversion of receiver chromosomes in F2 progeny from F1 master females (*y*^<ccN>^ *w*^a^ *N*^Ax16^/++; Cas9/+ ♀ X *w*^−^ ♂). Eye color was used to distinguish progeny receiving donor (*w*^a^ = orange eyes) vs. receiver (*w*^−^ = white eyes) chromosomes

Widely used CRISPR-Cas9-based gene editing approaches^8^ involve enzymatic cleavage of a sensitive allele and repair by copying information from an exogenously provided cut-resistant oligonucleotide or double-stranded DNA template^9,10^. With regard to germline editing, however, we reasoned that, in heterozygous individuals carrying two different alleles of a gene, it might be possible to repair a cleavage-sensitive allele with sequences provided by a cut-resistant allele present on the companion chromosome. This type of allelic-drive could supplement a gene-drive system that copies itself in one genomic location by adding a second guide RNA (gRNA) to the gene-drive cassette that directs selective cleavage of a non-preferred allele at a separate genomic site. Such a dual gRNA drive system (e.g., Fig. 1a, b) should result in efficient super-Mendelian inheritance of both the gene-drive element and the beneficial allelic variant via germline transmission when carrier individuals mate with recipient individuals bearing the undesired cleavage-sensitive allele.

Here we present experimental evidence for two forms of allelic-drive. The first, copy-cutting involves a Cas9-gRNA complex selectively cutting one allelic variant, followed by homology-directed repair (HDR)-mediated repair and replacement with a non-cleavable allele of the same gene provided in-trans. The second, and more generally applicable form of allelic-drive, which we refer to as copy-grafting, involves copying a short genomic interval that encompasses a favored allele in proximity to a gRNA cut site. In the case of copy-grafting, the favored allele is associated with neighboring sequences resistant to gRNA cleavage.

## Results

### Allele-specific Cas9-dependent cleavage

The X-linked *Drosophila Notch* (*N*) locus is particularly well suited for testing the concept of allelic-drive since both loss- and gain-of-function dominant alleles of this locus have been characterized. Loss-of-function *N*^−^ mutations, which are non-viable when homozygous in females (or hemizygous in males), produce dominant wing margin notching and thickened veins phenotypes in heterozygous females (Fig. 1c, black arrows), while homozygous viable dominant gain-of-function alleles, designated as *Abruptex* (*N*^Ax^, denoted as N* in the figures) generate a contrasting vein-loss phenotype (Fig. 1c, light blue arrows). We noticed that one previously identified *N*^Ax^ allele (Ax16) eliminates a PAM site present on the wild-type allele (Fig. 1b, pink box) resulting in a Gly → Arg amino acid substitution^11^ (Supplementary Fig. 6). We designed a gRNA (gRNA-N+) anchored by this PAM site to direct cleavage of the wild-type *N*^+^ allele, but not the *N*^Ax16^ allele (Fig. 1a, b). The gRNA-N+ was incorporated into a DsRed-marked gRNA-only “CopyCat” element (ccN) designed to insert into, and copy itself, at the closely linked *yellow* locus, which is located 2 centiMorgans distal to *Notch* relative to the centromere (Fig. 1a, Supplementary Fig. 4b). Since genomic insertion of the ccN CopyCat element disrupts the function of the *yellow* locus we denote this allele as *y*^<ccN>^ (see Methods for full allele nomenclature). We hypothesized that, in the presence of an unlinked source of Cas9, this DsRed marked *y*^<ccN>^ CopyCat element would copy itself at the *yellow* locus and might also result in super-Mendelian inheritance of the gRNA-insensitive *N*^Ax16^ allele via copy-cutting.

We first tested whether the *y*^<ccN>^ allele would efficiently copy itself, as well as the neighboring uncleavable *N*^Ax16^ allele, onto a wild-type (*y*^+^ *N*^+^) X chromosome (Fig. 1a) when combined with an autosomal source of Cas9-provided in-trans (Fig. 1d). F0 females (♀) bearing the *y*^<ccN>^ and *N*^Ax16^ alleles were crossed to wild-type males (♂) homozygous for a Cas9 source on the third chromosome to generate F1 *y*^<ccN>^ *N*^Ax16^/++; Cas9/+ ♀ progeny, which we hereafter refer to as “master females” (abbreviated MF in figures). In parallel, F0 ♀ were crossed to *w*^*–*^ **♂** (lacking Cas9), to generate control F1 females (*y*^<ccN>^ *N*^Ax16^/++; +/+ ♀), which we used to assess baseline Mendelian inheritance in the absence of Cas9-mediated drive. F1 master females (and control females) were crossed to wild-type (*y*^+^ *N*^+^) ♂ carrying a normal X chromosome (or the multiply inverted *FM7* balancer chromosome, which suppresses recombination, Fig. 1d). The resulting F2 progeny were then scored both for transmission of the DsRed-marked *y*^<ccN>^ element and the *N*^Ax16^ allele.

Transmission percentages for the *y*^<ccN>^ (DsRed+) and dominant *N*^Ax16^ alleles in F2 ♂ revealed highly biased inheritance of both alleles wherein 85.3% of these progeny were DsRed+ and yet a higher percentage (93.6%) were *N*^Ax16^ (Fig. 1e). Since loss-of-function null *N*^−^ alleles are non-viable in males, which carry only a single X chromosome, F2 ♂ were either *N*^Ax16^ or *N*^+^. Evidence that such lethal *N*^−^ alleles were indeed being generated as a result of imprecise DNA repair mediated by the non-homologous end joining (NHEJ) pathway is provided below. Additionally, consistent with the observation that nearly all F2 **♂** displayed a *yellow*^−^ mutant phenotype, sequence analysis of individuals from non-DsRed lines revealed that they all carried NHEJ-induced loss-of function mutations (Fig. 1d, Supplementary Fig. 2).

We also observed super-Mendelian inheritance of the DsRed-marked *y*^<ccN>^ element in F2 ♀ progeny (72.4%) but were unable to score *Notch*-related phenotypes with certainty due to a high degree of mosaicism in which wings often displayed a mixture of wild-type, gain-, and loss-of-function phenotypes (Supplementary Fig. 3). Such somatic mosaicism most likely results from the maternal perdurance of Cas9–gRNA complexes deposited into the egg, even in animals that did not inherit the Cas9-GFP transgene^3,5,12,13^. In order to circumvent this difficulty in scoring *Notch* phenotypes in F2 **♀**, we established 104 individual lines derived from single F2 females (selected for absence of the Cas9-GFP transgene), thus permitting unambiguous scoring of *N* phenotypes in subsequent generations (Fig. 1d). DNA was prepared from each isogenic line and PCR products from the *Notch* locus were sequenced to determine what alterations, if any, were present at the *Notch*-gRNA cleavage site (Supplementary Tables 2 and 3). In this latter analysis, we also identified DNA sequence polymorphisms located in an intron adjacent to the *N*^Ax16^ mutation (6.5 Kb upstream of the *N*^Ax16^ mutation) that unambiguously distinguished the donor *y*^<ccN>^ *N*^Ax16^ bearing chromosome from the recipient chromosome (Supplementary Fig. 4a). Results of the phenotypic and molecular analysis of the individual F2 ♀ lines are summarized in Fig. 1d (see Supplementary Table 2 and 3 for extensive analysis and sequence data). Overall, the findings parallel those for F2 ♂ as we observed super-Mendelian transmission of the DsRed-marked *y*^<ccN>^ element (75%) and yet greater inheritance of the *N*^Ax16^ allele (87.5%) among the female founder lines (Fig. 1d). Also, no *N*^−^ alleles were recovered, which in this case was surprising as such loss-of-function alleles generated by NHEJ would be expected to be viable in a heterozygous condition (see explanation below).

An important feature of the analysis of gRNA-induced events in the individual F2 **♀** lines was our ability to assign specific copying or non-copying outcomes at the *Notch* locus to donor vs. receiver chromosomes. In order to achieve the same end while analyzing larger numbers of progeny, we marked the donor chromosome with the tightly linked white-apricot (*w*^a^) allele (0.5 centiMorgans from *Notch*, Supplementary Fig. 4b), which causes an orange eye phenotype (i.e., *y*^<ccN>^ *w*^a^ *N*^Ax16^). F1 master females (*y*^<ccN>^ *w*^a^ *N*^Ax16^/*y*^+^ *w*^**–**^ *N*^+^; Cas9/+) were crossed to *w*^**–**^ males (Supplementary Fig. 4), and the resulting F2 individuals could be scored with ~ 99.5% precision for inheritance of donor (*w*^a^) vs. receiver (*w*^−^) chromosomes. Compiled results from ~20 such crosses conducted in parallel (Fig. 1f) reveal the same overall trend regarding the conversion frequencies observed in the F2 ♀ lines (Fig. 1d), with allelic conversion rates of *N*^+^ to *N*^Ax16^ averaging to 79.5%, a rate approximately a third higher than for copying of the *y*^<ccN>^ CopyCat element (~60%). This is particularly notable given that gRNA-induced cleavage events at the *yellow* locus (100% in our study—Supplementary Fig. 2) were consistently greater than at the *Notch* site, where 8–10% of progeny typically retained an unaltered wild-type target sequence (Fig. 1d, Supplementary Table 2, and Supplementary Fig. 5b).

**Fig. 2:**
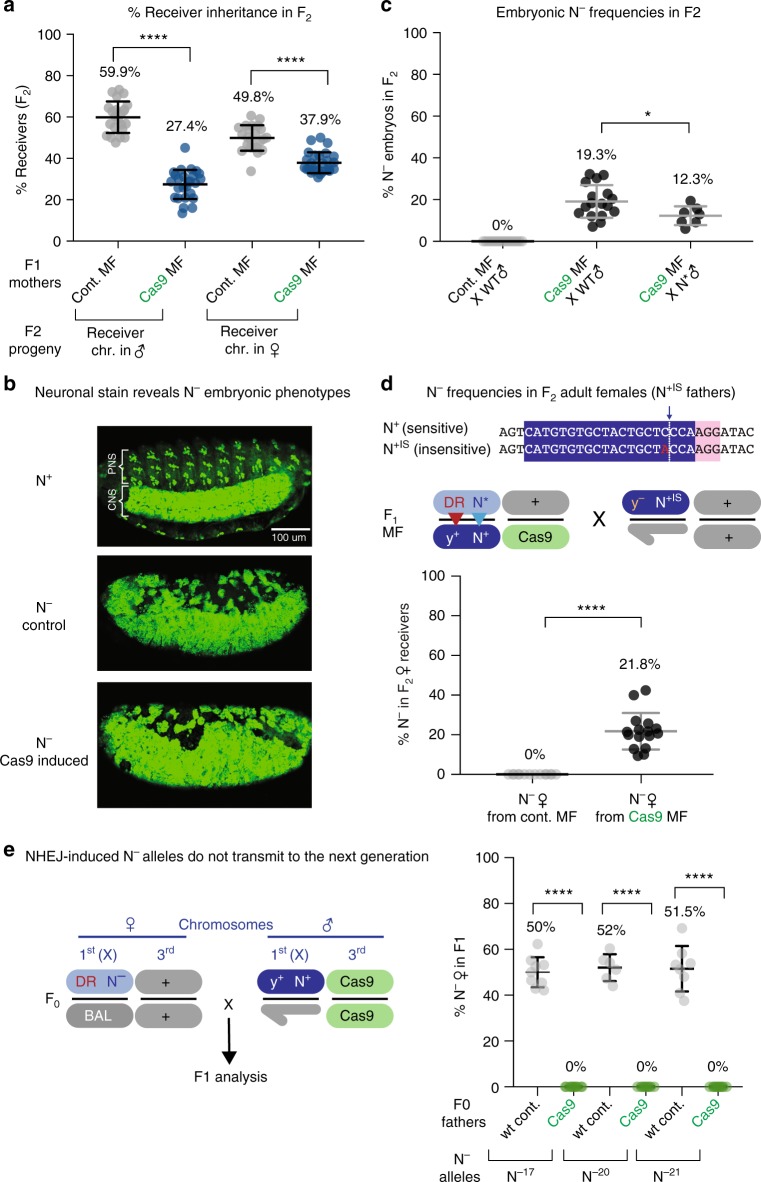
Drive-induced lethal events on the receiver chromosome. **a** The frequency of progeny inheriting the receiver chromosome from F1 master females (dark blue circles) compared to control F1 females lacking Cas9 (gray circles) in males vs. females. **b** Overnight embryo collections stained with an antibody against the pan-neural Elav protein. Embryos from wild-type females display normal central (CNS) and peripheral (PNS) nervous systems. Some embryos derived from F1 master females reveal a classic neurogenic phenotype in which nearly all cells derived from the ventral neuroectoderm develop as neurons. *N*^−^ control embryos collected from *N*^55e11/+^ mothers. **c** Frequencies of embryos displaying neurogenic phenotypes in collections derived from control *w*^−^ mothers or from F1 master females crossed either to wild-type (WT; actually *w*^−^) ♂ or to *N*^Ax16^ ♂. **d** Top panel: Sequence of a cleavage-insensitive *N*^+^ allele (*N*^+IS^). Note the nucleotide change from C → A at position −4 relative to the PAM sequence for the *N*^+IS^ allele (which results in the phenotypically silent S → Y amino acid substitution. Middle panel: Crossing scheme in which F1 master females are mated to males carrying a WT *N*^+IS^ allele. Other diagram elements are the same as in Fig. 1d. Lower panel: percentage of adult female progeny displaying dominant *N*^−/+^ heterozygous wing phenotype consisting of wing margin notches and thickened veins (see Fig. 1c). The presence of the *N*^−/+^ phenotypic class was strictly Cas9 dependent. **e** Left panel: crossing scheme for testing inheritance potential of an NHEJ-induced *N*^*−*^ allele by progeny of *y*^<ccN>^ *N*^−^/Balancer females crossed to males carrying a *vasaCas9* transgene on the third chromosome. Right panel: experimental results for three different NHEJ-induced *N*^−^ alleles (*N*^−17^, *N*^−20^, and *N*^−21^) revealing that zero progeny were recovered from ccN *N*^−^ females carrying any of these three *N*^**−**^ alleles in the presence of zygotically provided Cas9

We also examined the relative proportions of receiver vs. donor chromosomes in F2 progeny of F1 master females and observed a ~2-fold overabundance of donor chromosomes in males and a more modest, but highly statistically significant (*p* < 0.0001 in unpaired parametric *t* test analysis), parallel bias in females (Fig. 2a, blue circles). In contrast, among control crosses (for which no source of Cas9 was introduced), a greater proportion of male progeny inherited the wild-type (receiver) chromosome than the *N*^Ax16^ donor chromosome, presumably reflecting a fitness cost associated with the *N*^Ax16^ allele (Fig. 2a, gray circles). One potential explanation for the pronounced Cas9-induced reciprocal inheritance bias of the *N*^Ax16^ donor allele is that a fraction of gRNA-N+-induced cleavage events at the *Notch* locus on the receiver chromosome may result in NHEJ-induced *N*^−^ loss-of-function alleles. In males, such alleles would be hemizygous lethal resulting in strong embryonic neurogenic phenotypes causing much of the ventral epidermis to differentiate inappropriately as nervous system^14^. To test this hypothesis, we collected embryos from F1 master females (*y*^<ccN>^ *N*^Ax16^/++; Cas9/+ ♀) crossed to wild-type ♂ and observed an abundant class of mutants (~20%) with strong neurogenic phenotypes (Fig. 2b, c). As expected, neurogenic mutant embryos were absent from control crosses (i.e., from F1 *y*^<ccN>^ *N*^Ax16^/++ ♀, Fig. 2c).

### Dominant elimination of lethal NHEJ alleles

While the above results provide strong evidence for Cas9-dependent generation of *N*^−^ alleles in the progeny of F1 master females, they do not readily account for the failure to recover any heterozygous *N*^−^ alleles among the isogenic F2 ♀ lines. One possibility is that, if F2 ♀ progeny derived from F1 master females inherit a *N*^**−**^ allele from their mothers, the “wild-type” paternal allele might be acted on in a mosaic fashion by maternally perduring Cas9-gRNA complexes^3,5,12,13^. Such a maternal effect could result in a large enough proportion of somatic cells having two mutant copies of the *Notch* gene to preclude viability in heterozygotes. This hypothetical mechanism, which we refer to as lethal mosaicism, is consistent with the high frequency of mosaic wing phenotypes in F2 **♀** females we observed (Supplementary Fig. 3). One prediction of the lethal mosaic hypothesis is that, if F1 master females were crossed to males carrying the non-cleavable *N*^Ax16^ allele instead of wild-type males, the observed fraction of *N*^**−**^ F2 embryos should decrease significantly as a result of rescued lethality in females, and this was indeed the case (Fig. 2c).

Another avenue for testing the lethal mosaicism hypothesis was provided by sequencing our single F2 ♀ lines that inherited phenotypically wild-type *N*^+^ alleles (Supplementary Figs. 5b and 6). This analysis revealed that, while most of these “wild-type” *N*^+^ alleles matched the original native allele, one had point mutations in the gRNA core sequence (i.e., between 1 and 4 nucleotides from the PAM site). Such mutations would be predicted to result in cleavage-resistant or “insensitive” (*N*^+IS^) alleles (see Fig. 2d for sequence of specific *N*^+IS^ allele used below). As in the case of crossing master F1 females to *N*^Ax16^ ♂, we expected that crossing them to *N*^+IS^ ♂ (Fig. 2d) would also protect heterozygous female F2 progeny from lethal mosaicism since such individuals would carry one cleavage resistant functional *N*^+^ allele. The advantage of using *N*^+IS^ over *N*^Ax16^ fathers for such experiments is that *N*^**−**^/*N*^+IS^ ♀ heterozygotes are fully viable, whereas *N*^**−**^/*N*^Ax16^ ♀ exhibit significantly reduced viability. As predicted by the lethal mosaic hypothesis, a significant percentage (average = 22%; *p* < 0.0001 in unpaired parametric *t* test analysis) of F2 females derived from crosses of F1 master females to *N*^+IS^ ♂ displayed a typical heterozygous *N*^**−**/+^ wing phenotype in a Cas9-dependent fashion (Fig. 2d). Sequencing of several of such *N*^**−**^ alleles from individual F2 females revealed an array of DNA alterations centered at the cut site consisting of frameshifts, amino acid substitutions, and deletions (Supplementary Figs. 5a and 6). In addition, consistent with the embryo studies described above, from which we inferred that F2 females could be rescued from mosaic lethality by carrying a cleavage-insensitive *Notch* allele (Fig. 2c), we observed a 20% excess of females to males (*p* < 0.0001 in unpaired parametric *t* test analysis) among adult F2 progeny derived from crosses of master females to *N*^+IS^ ♂, which was not evident in parallel crosses to wild-type ♂ bearing the sensitive *N*^+^ allele (Supplementary Fig. 7).

An additional prediction of the lethal-mosaic hypothesis is that progeny inheriting the ccN drive element and any NHEJ-induced *N*^−^ allele from their mothers should be inviable if they also carried a zygotic source of Cas9. We tested this prediction by establishing three different lines in which NHEJ-induced *N*^−^ alleles (*N*^−17^, *N*^−20^, and *N*^−21^—Supplementary Fig. 5) were associated with the ccN element. When such F0 females (*y*^<ccN>^ *N*^−^/Balancer) were crossed to homozygous *vasaCas9* males (Fig. 2e), the only viable female F1 adult progeny recovered carried the *N*^+^ balancer (BAL) chromosome (Fig. 2e). The absence of emerging *N*^−^ F1 females in the Cas9 crosses contrasts with the expected 50% Mendelian transmission rate observed in non-Cas9 control crosses. These experiments demonstrate that NHEJ-induced *N*^−^ alleles, such as those created in the germline of master females, cannot be transmitted to the next generation in the presence of Cas9 since they produce fully penetrant dominant lethality in heterozygous animals carrying the ccN element.

As a yet more stringent test of the lethal mosaic hypothesis, we separately crossed both males and females carrying the ccN element combined with a wild-type cleavage sensitive *N*^+^ allele (*N*^+S^) to flies carrying the *vasaCas9* transgene. Again, no surviving F1 progeny inherited the *y*^<ccN>^ allele (Supplementary Fig. 8). We conclude that lethal mosaicism is a highly potent process that eliminates all progeny carrying the *y*^<ccN>^ allele, a hemizygous or homozygous cleavage-sensitive *Notch* allele, and a Cas9 source (either maternally or paternally provided). A corollary conclusion is that only progeny maintaining an association between the *y*^<ccN>^ and *N*^Ax^ alleles (or the very rarely generated *N*^+IS^ cleavage-insensitive alleles) can survive in the presence of Cas9.

**Fig. 3:**
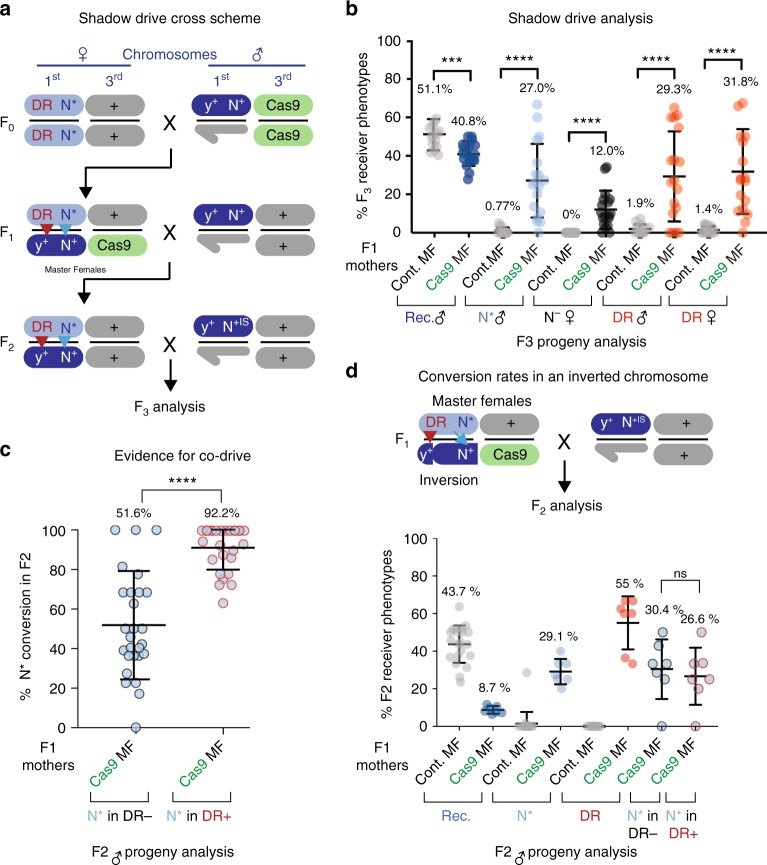
Shadow-drive and co-drive analyses. **a** Three-generation crossing scheme for analyzing shadow-drive in the F3 generation. F2 ♀ derived from F1 master females were crossed to *N*^+IS^ ♂, and F3 progeny were scored for percentage conversion of F2 receiver chromosomes and generation of *N*^−^ alleles. **b** Percentage of F3 progeny demonstrating features of drive including (from left to right): percentage of receiver chromosomes converted to *N*^Ax16^ allele (N*, blue circles; presence or absence of Cas9 refers to F1 generation); percentage of heterozygous *N*^−/+^ females (*N*^*−*^, black circles); and percentage of individuals having copied DsRed marked *y*^<ccN>^ element to receiver chromosomes in either males or females (red circles). The small percentages shown in control crosses (gray circles), in which F1 females lacked source of Cas9, presumably reflect the low rates of recombination between *w*^a^ and *N*^Ax16^ (0.5 cm) or the *y*^<ccN>^ element (1.5 cm). **c** Evidence for co-drive of *N*^Ax16^ (N*) allele with DsRed (DR+) marked *y*^<ccN>^ element among individuals inheriting receiver chromosomes. **d** Chromosome pairing is required for efficient allelic-drive and co-drive. Top panel: Genetic crossing scheme depicting allelic-drive to a balancer chromosome (Basc) that sustains approximately normal chromosome pairing for the *yellow* locus but not for *Notch*. Lower panel: Experimental results showing approximately normal levels of DsRed conversion of the Basc chromosome but significantly reduced copying (~1/3) of the *N*^Ax16^ allele. In addition, co-drive of the DsRed and *N*^Ax16^ alleles was abolished by the Basc inversion

The observation of pervasive somatic and lethal mosaicism in crosses of F1 master females to wild-type males raised the possibility that maternally inherited Cas9–gRNA complexes in F2 ♀ might also persist and act in the germline to generate some degree of gene-drive or allelic-drive in the F3 generation, even in animals that did not inherit the Cas9 transgene. We tested this possibility by crossing F2 *y*^<ccN>^ *N*^Ax16^/++;+/+ (non-Cas9) ♀ to *N*^+IS^ ♂ (Fig. 3a). F3 progeny from this cross did indeed manifest a substantial degree of perduring germline Cas9/gRNA activity as indicated by several measures (Fig. 3b), including: (1) copying of the DsRed-labeled *y*^<ccN>^ element onto the *y*^+^*N*^+^ receiver chromosome (29.3% and 31.8% conversion of the receiver chromosome in males and females, respectively), (2) copying of the *N*^Ax16^ allele (27% conversion of the receiver chromosome in males), (3) recovery of *N*^−^/*N*^+^ ♀ (12%), and (4) Cas9-dependent depletion of receiver chromosomes in males (40.8% compared to 51.1% in control animals). Each of these measures of residual drive observed in the F3 generation (Fig. 3b), which we refer to as shadow-drive, was considerable, amounting to roughly half of that observed in the prior F2 generation.

### Co-transmission of a protected allele and drive element

One surprise emerging from analysis of the copying efficiency of the *y*^<ccN>^ CopyCat element vs. the *N*^Ax16^ allele, which was also a striking trend in the 104 single female lines (Fig. 1d), was an unexpected high positive correlation between these two conversion events. In nearly all cases where the CopyCat element was copied to a receiver chromosome, so too was the *N*^Ax16^ allele. This co-drive phenomenon can be most readily appreciated by considering the fraction of *N*^Ax16^ conversion events among DsRed+ (*y*^<ccN>^) receiver chromosomes (92.2%), which is nearly double that of *N*^Ax16^ conversion among receiving chromosomes that failed to copy the *y*^<ccN>^ element (51.6%) (Fig. 3c). Similarly, among the single F2 female lines, 96% of the receiver chromosomes carrying the DsRed+ *y*^<ccN>^ element also copied the *N*^Ax16^ allele, in contrast to only 38% of progeny having copied the *N*^Ax16^ allele to a receiver chromosomes in the absence of *y*^<ccN>^ copying (Fig. 1d). We examined whether this co-drive phenomenon depended on chromosome pairing by placing the *y*^<ccN>^ *N*^Ax16^ chromosome in-trans to a multiply inverted balancer X chromosome (Basc). This chromosomal arrangement resulted in a loss of co-drive as well as a marked reduction in the frequency of *N*^Ax16^ copying (Fig. 3d). It is notable that the frequency of DsRed copying in these crosses was only slightly reduced relative to that observed with a wild-type receiver chromosome, consistent with the tip of the Basc chromosome, which includes the *yellow* locus, being co-linear with wild-type chromosomes (note: the Basc 1B3 inversion breakpoint is located between *y* and *N*). We also tested whether co-drive of the *N*^Ax16^ allele depended on active copying at the *yellow* locus by placing the *y*^<ccN>^ *N*^Ax16^ chromosome in-trans to a receiver chromosome carrying an NHEJ-induced point mutation at the y-gRNA cleavage site. In this “single cut” configuration, we observed reduced transmission of the *N*^Ax16^ allele relative to that in control crosses using a wild-type (*y*^+^*N*^+^) receiver chromosome (Supplementary Fig. 9). This latter experiment indicates that copying the *y*^<ccN>^ element increases allelic-drive at the *N* locus. Finally, we examined the impact of cis- vs. trans- configurations for the *y*^<ccN>^ and *N*^Ax16^ alleles by generating master females of the genotype *y*^<ccN>^ *N*^+S^/*y*^+^ *N*^Ax16^; Cas9/+ (Supplementary Fig. 10) and observed efficient drive of both the DsRed-marked *y*^<ccN>^ element (76% conversion) and the *N*^Ax16^ allele (93% conversion) indicating that both cis- and trans- configurations of the two elements sustain potent drive.

While copying of the *Ax16* allele from the donor to the receiver chromosome was the overwhelmingly prevalent event in the above allelic-drive experiments, we also identified rare in-frame NHEJ-induced indels induced at the gRNA-N+ cut site that generated de novo *Abruptex* alleles (Supplementary Figs. 5c and 6). We tested whether it was possible to produce allelic-drive with one such de novo allele (*Ax103*) by recombining *N*^Ax103^ with the *y*^<ccN>^ element. We found that it performed equivalently to the *N*^Ax16^ allele in driving conversion of a wild-type *N*^+^ allele to an Abruptex phenotype (Supplementary Fig. 11). This finding demonstrates that allelic variants with mutations  at the Cas9 cleavage site are equally well suited as those disrupting a PAM site for sustaining efficient allelic-drive.

### Copy-grafting is a broadly applicable allelic-drive strategy

The experiments described above demonstrate highly efficient allelic-drive of *N*^Ax^ alleles via copy-cutting mechanism, exceeding by nearly a third that observed for the *y*^<ccN>^ gene-drive CopyCat element. While these results are encouraging, the obvious limitation of such a strategy lies in the requirement for a gRNA to selectively cut the targeted undesired allele. This constraint requires that the preferred allele either lacks a PAM site or differs from the targeted allele in core gRNA sequences (~1–5 nucleotides from the PAM site), which would occur in only a fraction of cases (~60% of single-nucleotide polymorphisms if GG di-nucleotides occur at a frequency of 1/16 and are randomly distributed). We speculated that it might be possible to develop a more general allelic-drive method by making use of the fact that HDR is often accompanied by local gene conversion events spanning as much as several hundred nucleotides from the double-stranded cleavage site^15^. This local repair phenomenon has been well documented in *Drosophila*^16–18^ and may reflect the range of 3′ resection during the DNA repair process^16^.

**Fig. 4:**
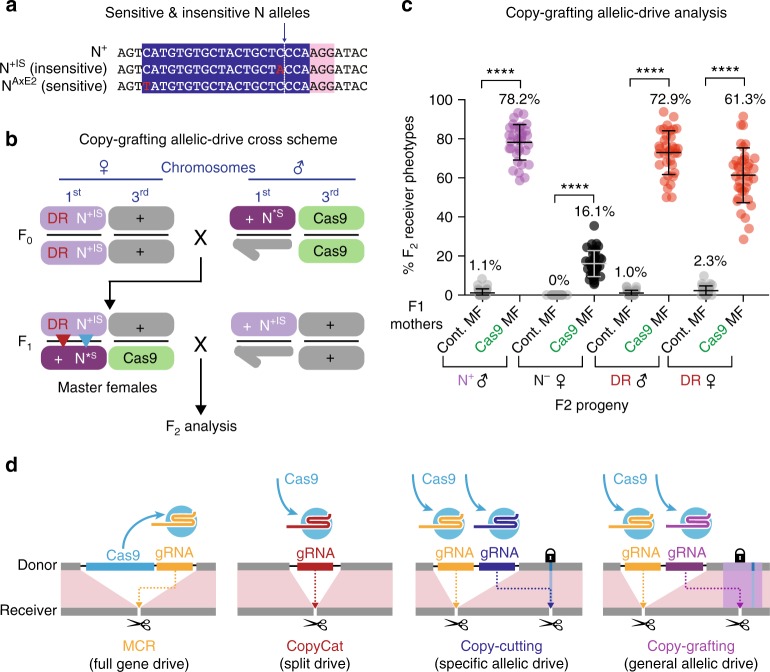
Allelic-drive mediated by “copy-grafting.” **a** DNA sequence at the gRNA-N+ cleavage site for the wild-type reference allele (*N*^+S^), the cleavage-insensitive wild-type (*N*^+IS^), and the cleavage-sensitive *N*^AxE2^ allele, with the C → T substitution (see Supplemental Fig. 6 for amino acid substitutions). **b** Copy-grafting scheme in which F1 master females carrying a wild-type cleavage insensitive wild-type *Notch* allele in trans to the sensitive *N*^AxE2^ allele (N*^S^) (*y*^<ccN>^ *w*^a^ *N*^+IS^/*y*^+^ *w*^+^ *N*^AxE2^; Cas9/+ ♀) are crossed to *N*^+IS^ ♂. F2 progeny were then scored based on inheritance of the *y*^<ccN>^ *w*^a^ *N*^+IS^ donor chromosome (light purple) or the *w*^**+**^ receiver chromosome (dark purple) based on their eye color phenotype (orange for donor and red for receiver). Red and blue arrowheads indicate copying of the *y*^<ccN>^ element and the *N*^+IS^ allele, respectively. **c** Percentage of F2 progeny demonstrating features of drive including: percentage of converted *N*^+IS^ receiver chromosomes (purple circles); heterozygous *N*^−/+^ females (black circles); and copying of the DsRed marked *y*^<ccN>^ element to receiver chromosome (red circles). **d** Summary of different drive systems. MCR (full gene drive) elements have both Cas9 (blue) and a gRNA (yellow) inserted into the genome at the gRNA-directed cleavage site. CopyCat (split drive) elements carry only the gRNA (red) inserted at the gRNA-directed cleavage site, while Cas9 is provided from a Mendelian transgene at a different genomic location. Copy-cutting (special allelic-drive) is mediated by two gRNAs. One gRNA (yellow) propagates the CopyCat element while the second gRNA creating allelic-drive (dark blue) cuts a non-preferred allele, but not the favored allele (lock icon). This drive element could be either an MCR (including Cas9) or a CopyCat element (Cas9 provided from a separate genomic location, as depicted in this figure). Copy-grafting (general allelic-drive) is mediated by a gRNA (purple) that cuts the non-preferred, but not the favored, chromosome near the desired allelic variant, resulting in conversion of a short region of the receiving chromosome (indicated by purple highlight) with sequences from the donor chromosome (purple box) that encompass the favored allele (lock icon)

With the above motivating concept in mind and the rich array of genetic variants available in *Drosophila*, as well as those we generated in this study, we conceived of a reverse-drive scenario wherein the wild-type *N*^+IS^ allele should be preferentially inherited over a different cleavage-sensitive, *Abruptex* allele (*AxE2*). The *N*^AxE2^ allele results from a C → T substitution located 21 bp upstream of the cleavage-resistant *N*^Ax16^ G → A alteration (Fig. 4a). The *N*^AxE2^ allele, however, should be sensitive to cleavage by the gRNA-N+ carried on the *y*^<ccN>^ CopyCat element since it lies at a sufficient distance from the gRNA cut site (17 bp, Fig. 4a, *N*^AxE2^ indicated as N*^S^ in Fig. 4b). We reasoned that, if the *y*^<ccN>^ CopyCat element were recombined with the wild-type *N*^+IS^ cleavage-insensitive allele, which carries a single-nucleotide change (C → A) at the −4 position (Fig. 4a), it might be possible to drive that wild-type *N* allele onto a receiving chromosome carrying the *N*^AxE2^ allele. This inverse-drive scheme depicted in Fig. 4b is referred to as copy-grafting.

Results in Fig. 4c reveal that the efficiency of inverse allelic-drive via copy-grafting (78.2% conversion of receiver chromosomes in males) was comparable to that observed by copy-cutting (79.5% conversion—Fig. 1f). Also, as we observed for copy-cutting, copy-grafting resulted in a dearth of receiver chromosomes in F2 ♂ progeny, in the generation of *N*^**−**^ alleles (~16%; when F1 master females were crossed to *N*^+IS^ ♂ to protect the paternal chromosome from lethal mosaicism), and in strongly correlated co-drive between the *y*^<ccN>^ and *N*^AxE2^ alleles (Supplementary Fig. 12).

## Discussion

In this study, we have demonstrated the feasibility of two forms of allelic-drive: copy-cutting, which applies to cases in which a gRNA can be designed to selectively target a non-preferred allele; and copy-grafting, a more general strategy in which one associates a cleavage-resistant site in proximity to a favored allelic variant (Fig. 4d). Both allelic-drive systems were significantly more efficient than a gene-drive CopyCat element inserted into the *yellow* locus. An important mechanism contributing to the efficient allelic-drive we observed, which is of potential relevance to field applications, is the phenomena of lethal mosaicism, which dominantly eliminates all NHEJ-induced drive-resistant non-functional loss-of-function alleles of an essential gene such as *Notch*. Shadow-drive, in which perduring Cas9–gRNA complexes transmitted maternally for one generation in the absence of the Cas9 or gRNA transgenes, should also act as a genetic slingshot to extend gene- or allelic-drive for one additional generation should they become separated from a Cas9 source. Interestingly, we observed a strong correlation between copying events resulting in preferential transmission of both the gene-drive cassette and the preferred non-cleavable allele. This co-drive phenomenon depends on chromosome pairing, as it is not observed when the receiver chromosome carries an inversion affecting the targeted gene. We note that similar correlated genome editing events have been reported recently in other systems including in the germline^19^ and in somatic cells^20^. With regard to copy-cutting, the recent development of Cas9 variants with broadened PAM specificity^21,22^ may substantially increase the fraction of alleles that can potentially be driven by this method. Development of such systems should function with various sources Cas9, as we have also observed allelic-drive with a *nanos*Cas9 transgene as efficiently as with *vasaCas9* (Supplementary Fig. 13). Altogether, our study suggests that allelic-drive strategies are likely to be broadly applicable to various gene targets in diverse experimental and agricultural contexts.

One important category of potential applications active genertics is the aggregation of multiple favored naturally occurring allelic variants in plants or animals. In plants, allelic-drive schemes could facilitate combining favorable traits to improve crop yields and resistance to environmental stresses, particularly in polyploid species (e.g., wheat or rye). Similarly, in animal models, allelic-drives could accelerate the construction of complex genotypes for biomedical and basic research. For example, one could envision crossing individual plants or animals bearing a favored allele to a strain carrying a Mendelian cassette with Cas9 and a gRNA that target the corresponding non-preferred alleles. The Cas9-bearing progeny also inheriting the first favored allele could then be crossed to a strain carrying the second favored allele and a gRNA targeting the second unwanted allele and so on until all favored alleles are gathered into a single strain. Finally, one could perform a final cross to segregate out the Cas9-gRNA cassettes. In polyploid crops, such a strategy should permit assembly of several preferred alleles providing drought resistance^23^, higher yields^24^, optimal architectures^25^, or more rapid growth^26^ that would be long, difficult, or impossible to assemble into a single strain by standard genetic crossing schemes.

Another potentially important class of allelic-drive applications would be to reverse pesticide resistance in pest species. Use of insecticides has repeatedly led to the emergence of specific insecticide-resistant alleles in insect disease vectors and crop pests. Many pesticides target essential components of the nervous system such as the Na^+^ channel or glutamate receptor^27^. In principle, allelic-drive systems could help revert these populations back to their wild-type-sensitive state, which would be aided by the reduced fitness of certain prominent insecticide-resistant alleles in the absence of pesticide use^28–30^. It is worth noting that even modest reductions in the incidence of resistant alleles (e.g., to prevalence of <50%) can have major positive impacts on disease-reduction strategies^31,32^. Similarly, allelic-drives could be used to favor genetic variants that prevent host species from serving as disease vectors or pests.

One potential concern in such allelic-drive scenarios is whether NHEJ-induced cleavage-resistant alleles could also be driven by the gRNA intended to drive the favored allele. This run-away NHEJ problem might arise if the primary drive cassette became separated from the preferred allele and instead became associated with an undesired NHEJ-induced allele. Several lines of evidence presented in the current study suggest that this scenario is unlikely so long as the gRNA sustaining allelic-drive targets a critical region of an essential gene such as *Notch* (or the Na^+^ ion channel). First, the strong co-drive we observed greatly limits the number of events separating the favored allele from the drive element (only a few percent in our experiments). Second, lethal mosaicism eliminated 100% of the progeny carrying three different NHEJ-induced *N*^−^ alleles and also killed all offspring carrying the gene-drive element and unprotected wild-type *N*^+^ alleles. Finally, allelic-drives work very effectively in-trans as well as in-cis, indicating that, should an uncoupling event occur, it would be rapidly reversed in most instances. Thus all non-functional NHEJ alleles will be eliminated immediately as they are generated, and drive of the favored allele will persist either in-cis or in-trans. The only remaining concern is whether one might not occasionally create a functional non-cleavable version of the undesired allele that could then also be driven. While this is possible (e.g., we did recover rare *N*^+IS^ and novel *Ax* alleles), such events are infrequent and should not drive any more efficiently than the overwhelmingly prevalent preferred allele. It may be possible to further reduce the production of such rare events by using two gRNAs simultaneously, one directing copy-cutting and the other copy-grafting of the same preferred allele. Thus it should be practical to drive preferred alleles of essential genes efficiently into a population so long as they do not impart a significant fitness cost relative to the non-preferred allele.

Lethal mosaicism should also have game-changing implications for developing new efficient gene-drive systems. A drive element targeting a critical site in a gene essential for viability or reproduction could also carry a functional recoded (and non-cleavable) portion of that same gene, thereby protecting progeny inheriting this element from lethal (or sterile) mosaicism. In contrast, non-functional NHEJ-induced mutations rendered dominant by lethal mosaicism should be eliminated immediately, thereby “killing or sterilizing the mistakes” and providing a powerful solution to the frequently highlighted drive-resistance problem. These and other diverse applications of allelic-drive should greatly expand the impact of active genetics, accelerating progress in many areas of synthetic biology.

## Methods

### Construction of ccN CopyCat element

Cloning of the ccN CopyCat plasmid followed the same strategy as described in Xu et al.^7^ using homology arms to the *yellow* locus abutting gRNA-y1 cleavage site and carrying gRNA-y1, gRNA-N+, and a 3XP3-DsRed eye marker as depicted in Supplementary Fig. 1 (the full DNA sequence of this plasmid is provided in Supplementary Table 1). Following assembly of its components, the ccN CopyCat plasmid was transformed into One Shot® TOP10 competent cells (Invitrogen #C4040) and purified using the Qiagen Plasmid Midi Kit (#12191). An injection mix containing the ccN plasmid (final concentration: 250 ng/µl) was sent to Best Gene Inc. for injection into embryos collected from a *w*^a^ *N*^Ax16^ *rb*^−^ stock (which is resistant to the otherwise lethal mutagenesis of the *Notch* locus generated by Cas9/gRNA-N+) with a transient source of pHsp70-Cas9 (Addgene plasmid #45945). The *w*^a^ *N*^Ax16^ *rb*^−^ stock was kindly provided by Jim Posakony (UCSD). Male transformants carrying the ccN element were identified in F1 progeny by virtue of their *yellow*^−^ and DsRed fluorescent eye-marker phenotypes. This genomic insertional allele is referred to as: *y*^<CC|gRNA-y1, gRNA-N+|3XP3DsRed>^ in accordance with our previously established nomenclature convention^6,7^ (see Supplementary Fig. 1 for details of the construct). For shorthand in the text, we refer to this allele as: *y*^<ccN>^. The ccN plasmid construct was fully sequenced prior to injection as well as that of the ccN genomic insertion for several individual *y*^<ccN>^ transformant lines, which included PCR amplification and sequencing of endogenous sequences lying adjacent to those included as homology arm templates in the plasmid construct to verify accurate insertion of the ccN element into the intended site (Supplementary Table 1).

#### Genomic DNA preparation

Genomic DNA from single adult flies were prepared according to protocols by Gloor et al.^33^. Single flies were crushed in lysis buffer (10 mM Tris pH8.2, 1 mM EDTA, 25 mM NaCl, with 0.3 mg/ml Proteinase K, added right before incubation), incubated at 37 °C for 30 min, and heated at 95 °C for 2 min. One hundred μl of ddH_2_O were added to each tube before storage at −20 °C.

#### Drosophila genetics

Flies carrying the donor *y*^<ccN>^ *w*^a^ *N*^Ax16^ chromosome were identifiable through the visible *w*^a^ orange eye phenotype. *y*^<ccN>^ *w*^a^ *N*^Ax16^/FM7 females were crossed to *vasaCas9* homozygous males (BL# 51324) to generate F1 master females as diagrammed in Fig. 1d. Crosses were performed at 25 °C on standard *Drosophila* food. The *y*^−^ w^+^ *N*^AxE2^ line carrying a gRNA-N+-sensitive *Abruptex* allele was kindly provided by Spyros Artavanis-Tsakonas (Harvard University). A cleavage-insensitive *N*^+^ allele recovered among the 104 isogenic lines was recombined with *y*^<ccN>^ *w*^a^ to generate the donor chromosome in Fig. 4b. For quantitative analyses of F2 progeny (or F3 progeny for the shadow-drive), 20–37 crosses consisting of 3 females mated to 3 males were analyzed for each experiment, yielding an average of ~150 flies per cross. A total of 29,000 progeny were analyzed for this study.

#### Sequence analysis

To sequence mutations in the *yellow* locus, an ~500-bp fragment was amplified by PCR (Q5 Hot Start High-Fidelity 2× Master Mix) with primers 417 (TTTAGTGCCTCAATAATAGTTTGGCCCTGC) and 356 (GGACATACCAAATATACCCTCC), then sequenced with primer 418 **(**GGAAGTTAATACCAGCGACATTGAAATCGC) at Genewiz. To identify donor vs. receiver chromosomes, a fragment from Notch intron 5 was amplified with primers NintS3 (CTACGAGTGCAAGTGCCCCAAAG) and NintAS3 (CGCCCGGAACGTTGGAATGGAATG) and sequenced with NintS3bis (CAGTAGGAACCAGATTAATCGAGTT). For sequencing mutations in the *N*^Ax^ region, primers NAxS (CCACGAGCAAAACAACGAGTACAC) and NAxAS2 (TTCGAATCACAATCCTGACCACTCAGC) were used to amplify an ~1-Kb fragment and sequenced using primer NAxS3 (GCATCAATGGCTACAACTGTAGC).

#### Active genetic safety measures

All crosses using active genetics were performed in accordance to an Institutional Biosafety Committee-approved protocol from UCSD in which full gene-drive experiments are performed in a high-security ACL2 barrier facility and split drive experiments are performed in an ACL1 insectary in plastic vials that are autoclaved prior to being discarded in accord with currently suggested guidelines for laboratory confinement of gene-drive systems^34,^^35^.

#### Wing dissection and mounting

*Drosophila* wings were dissected in isopropanol and mounted in 100% Canada balsam.

#### Antibody staining of *Drosophila* embryos

Fixation and antibody staining of embryos using a rat anti-Elav (DSHB# 7E8A10, antibody dilution = 1/20) was performed according to standard procedures. Samples were mounted in Slowfade diamond anti-fade mountant (Thermo Fisher Scientific #S36963) and imaged on a Leica SP5 confocal microscope. Each data point in Fig. 2c corresponds to the analysis of a group of 30–50 embryos on a slide. Embryos of stages 11–16 were scored for *N*^+^/*N*^−^ phenotypes using a Zeiss AXIO ZOOM V16 fluorescent microscope.

#### Ethical conduct of research

We have complied with all relevant ethical regulations for animal testing and research and conformed to the UCSD institutionally approved biological use authorization protocol (BUA #311).

### Reporting summary

Further information on experimental design is available in the Nature Research Reporting Summary linked to this article.

## Supplementary information

Supplementary Information

Reporting Summary

Source Data

## Data Availability

All relevant data are available from the authors.
