# Supplementary material for: Efficient allelic-drive in Drosophila
Source: Nat Commun. 2019 Apr 9;10:1640. doi: 10.1038/s41467-019-09694-w (PMC6456580; doi:10.1038/s41467-019-09694-w)
Supplement: Supplementary file 1 — Supplementary Information [file 41467_2019_9694_MOESM1_ESM.pdf]

Supporting Online Material

Supplementary Figures and Tables

Supplementary Figure 1

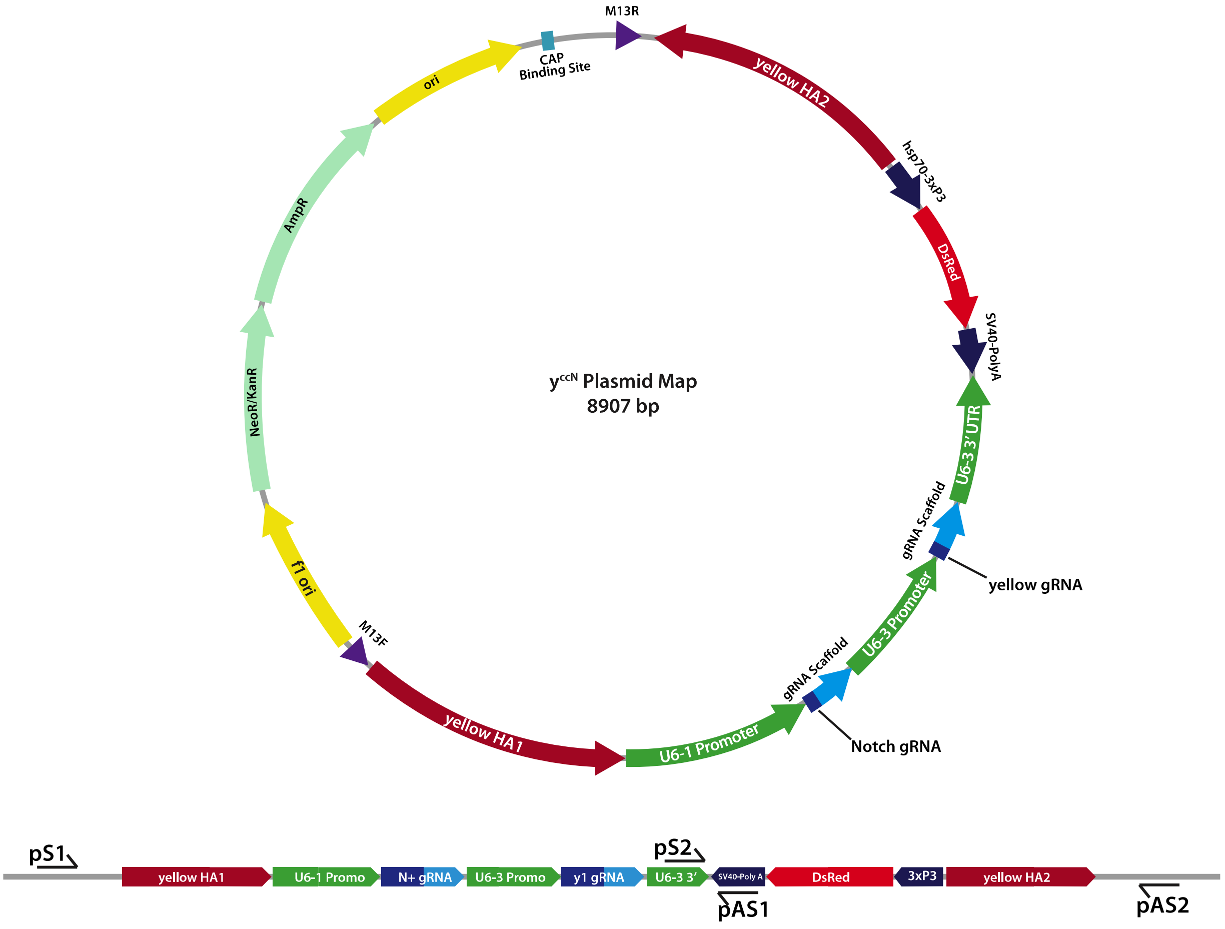

Supplementary Figure 1: Top panel: Circular map of ccN CopyCat element plasmid indicating the location of the homology arms for insertion into the yellow locus (*yellow* HA1, 2), a *yellow* guide RNA for insertion into and for copying at the *yellow* locus, a gRNA for cutting the wild-type Notch locus but not Ax16 allele (gRNA-N+ in text), and a DsRed fluorescence marker. Bottom panel: Linear map showing integration of the ccN element into the *yellow* locus and the locations of primers used to PCR amplify fragments for sequencing. Insertion of the ccN element into its target site and its flanking genomic sequences were amplified using primers

located beyond (~300bp) the HA1 and HA2 arms (pS1: CGACTCAGCTCACGTATTTCATACAG with pAS1: CTCCCCCTGAACCTGAAACATAAAATG, and pS2:CAGGAGCTCCAGCTTTTGTGCTAG with pAS2:GAGAATATACCCAAGTACCGTGTGTAG) and fully sequenced, which confirmed a perfect insertion of the element into the intended genomic target site. Sequences within the ccN element were identical to those in the vector shown in Supplementary Table 1. The full nomenclature for the ccN CopyCat element according to our previously established convention for active genetic elements [1,2] is: $y^{\text{<CC|gRNA-y1, gRNA-N+|3XP3DsRed>}}$, which we abbreviate in the text as $y^{\text{<ccN>}}$ for convenience. The complete DNA sequence of the ccN element is provided in Supplementary Table 1.

Supplementary Figure 2

				Base Changes	
Rec.	AAGTGGATGAGTGTGGTCGGCTGTGGGTTTTGGACACTGGAA		CCGTGGGCATCGGCAATACCACCACTAATCCGTGCCCC	WT	
55	AAGTGGATGAGTGTGGTCGGCTGTGGGTTTTGGACACTGGA·		CCGTGGGCATCGGCAATACCACCACTAATCCGTGCCCC	−1	
62	AAGTGGATGAGTGTGGTCGGCTGTGGGTTTTGGACACTGGA·		CCGTGGGCATCGGCAATACCACCACTAATCCGTGCCCC	−1	
70	AAGTGGATGAGTGTGGTCGGCTGTGGGTTTTGGACACTGGA·		CCGTGGGCATCGGCAATACCACCACTAATCCGTGCCCC	−1	
53	AAGTGGATGAGTGTGGTCGGCTGTGGGTTTTGGACAC·····		·CGTGGGCATCGGCAATACCACCACTAATCCGTGCCCC	−6	
37	AAGTGGATGAGTGTGGTCGGCTGTGGGTTTTGGAC·······		·CGTGGGCATCGGCAATACCACCACTAATCCGTGCCCC	−8	
44	AAGTGGATGAGTGTGGTCGGCTGTGGGTTTTGGAC·······		·CGTGGGCATCGGCAATACCACCACTAATCCGTGCCCC	−8	
36(5X)	AAGTGGATGAGTGTGGTCGGCTGTGGGTTTTGGACACTGGA·	CA	CCGTGGGCATCGGCAATACCACCACTAATCCGTGCCCC	−1	+2
79	AAGTGGATGAGTGTGGTCGGCTGTGGGTTTTGGACACTG···	TGG	········ATCGGCAATACCACCACTAATCCGTGCCCC	−11	+3
11(2X)	AAGTGGATGAGTGTGGTCGGCTGTGGGTTTTGGACACTGG··	GCAGATG	CCGTGGGCATCGGCAATACCACCACTAATCCGTGCCCC	−2	+7

Supplementary Figure 2: Sequence analysis of Cas9-induced *yellow*- mutations on receiver chromosomes. DNA was isolated from *y*- DsRed- isogenic lines described in Supplementary Table 2 and sequenced for NHEJ-induced mutations at the gRNA-y1 cleavage site. DNA sequences of several such alleles are presented, with in-frame y^{-} alleles highlighted.

Supplementary Figure 3

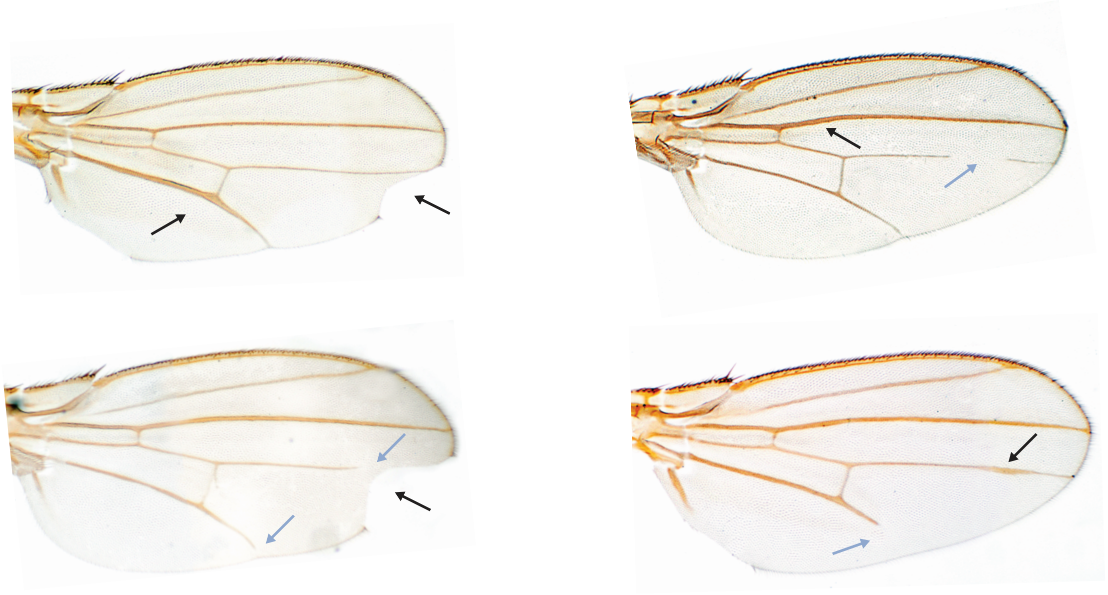

Supplementary Figure 3: Mosaic phenotypes of F2 female progeny of master females. Examples of mosaic wing phenotypes observed in female F2 progeny of master females depicted in Fig. 1d. Note the combination of phenotypes typical of Notch loss-of-function alleles consisting of wing margin notches and thickened veins (black arrows) and gain-of-function Abruptex alleles typified by distal vein truncations or gaps, which are particularly prominent in the L4 and L5 veins (blue arrows).

Supplementary Figure 4

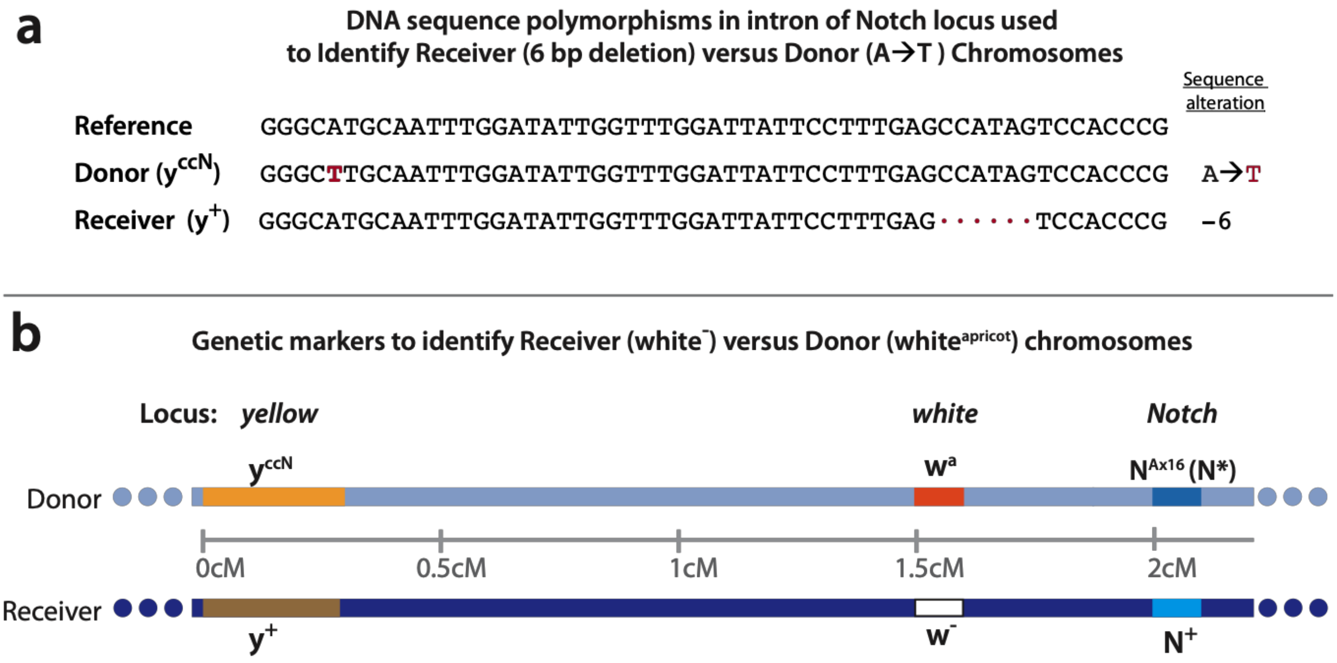

Supplementary Figure 4: Molecular and genetic markers for scoring donor versus receiver chromosomes.

a) Molecular markers: Notch intron DNA sequence polymorphisms used to score donor (A→T) versus receiver (6 nt deletion) chromosomes.

b) Genetic markers: Alleles of the *white* locus, which is closely linked to Notch (0.5 centimorgans) were used to distinguish individuals carrying donor (w^a = orange eyes) from receiver (w^- = white eyes) chromosomes.

Supplementary Figure 5

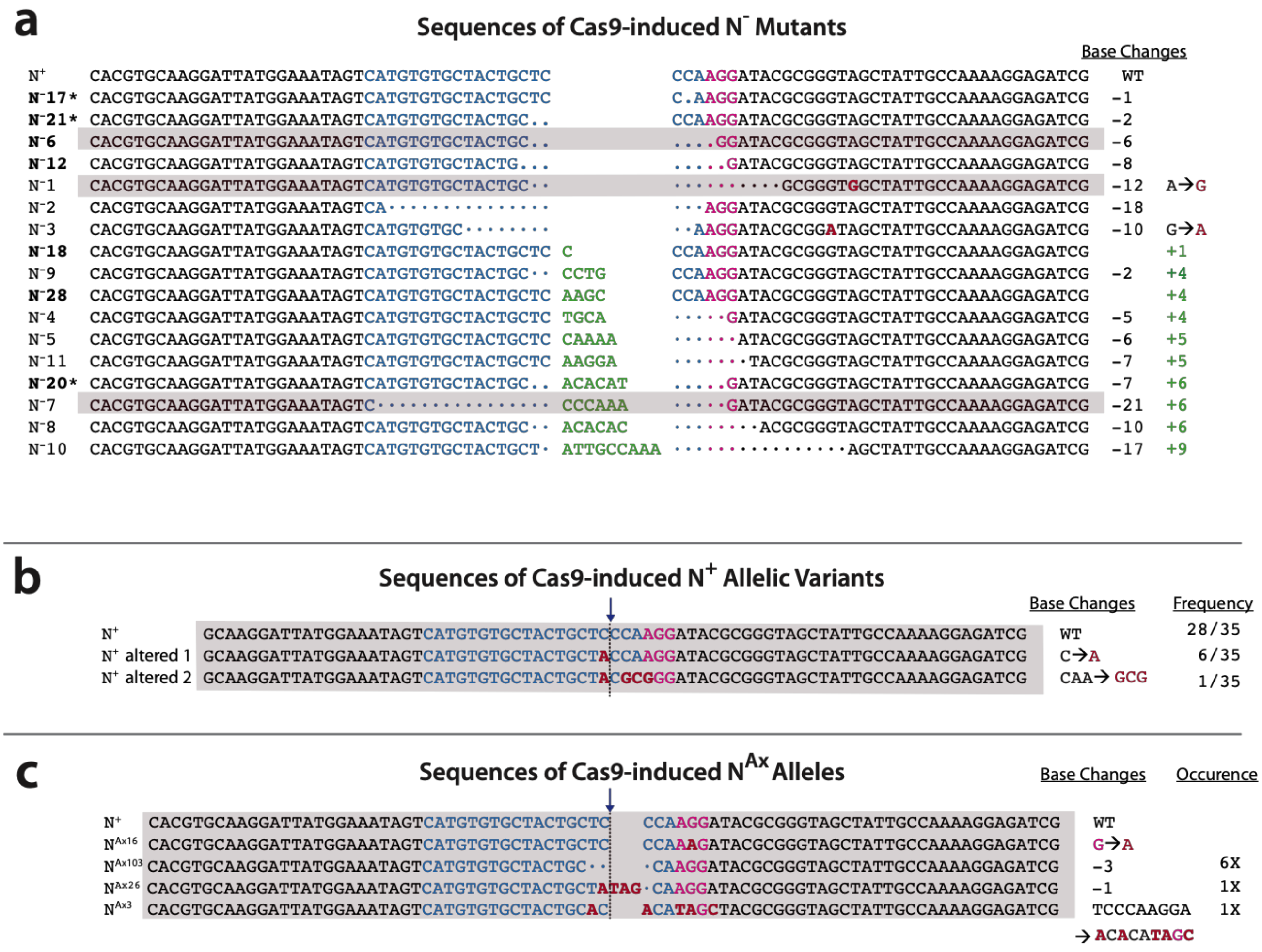

Supplementary Figure 5: Sequence analysis of Cas9-induced *Notch* mutations and allelic variants.

a) Loss of function *Notch* alleles. Highlighted sequences are in-frame indels. **b)** *Notch* alleles inherited from master females with wild-type function. Note that out of 35 such alleles sequenced (derived from isogenic females plus additional independently generated wild-type alleles): 28 matched that of the original receiver chromosome, 6 independent mutations were recovered in with a C→A mutation at the -4 position relative to the PAM sequence (e.g., immediately 5’ to the gRNA cut site (indicated by

dashed vertical line), and one compound mutant with a C->A mutation at the -4 position and CA→GC alterations at the -2, -3 positions. **c)** NHEJ-induced Abruptex alleles. The TCC deletion at position -2 -1 +1 was recovered 6 independent times, while two other Ax mutations were recovered only once.

Supplementary Figure 6

N^{+} Rec.	AGT	CAT	GTG	TGC	TAC	TGC	TCC	CAA	GGA	TAC	GCG	GGT	AGC	TAT		
	S	H	V	C	Y	C	S	Q	G	Y	A	G	S	Y		
N^{+IS1}	AGT	CAT	GTG	TGC	TAC	TGC	TAC	CAA	GGA	TAC	GCG	GGT	AGC	TAT		
	S	H	V	C	Y	C	Y	Q	G	Y	A	G	S	Y		
N^{+IS2}	AGT	CAT	GTG	TGC	TAC	TGC	TAC	GCG	GGA	TAC	GCG	GGT	AGC	TAT		
	S	H	V	C	Y	C	Y	A	G	Y	A	G	S	Y		
N^{Ax16}	AGT	CAT	GTG	TGC	TAC	TGC	TCC	CAA	AGA	TAC	GCG	GGT	AGC	TAT		
	S	H	V	C	Y	C	S	Q	R	Y	A	G	S	Y		
N^{AxE2}	AGT	TAT	GTG	TGC	TAC	TGC	TCC	CAA	GGA	TAC	GCG	GGT	AGC	TAT		
	S	Y	V	C	Y	C	S	Q	G	Y	A	G	S	Y		
$N^{Ax103/11}$	AGT	CAT	GTG	TGC	TAC	TGC	···	CAA	GGA	TAC	GCG	GGT	AGC	TAT		
	S	H	V	C	Y	C	–	Q	G	Y	A	G	S	Y		
N^{Ax26}	AGT	CAT	GTG	TGC	TAC	TGC	TAT	AGC	CAA	GGA	TAC	GCG	GGT	AGC	TAT	
	S	H	V	C	Y	C	Y	S	Q	G	Y	A	G	S	Y	
N^{Ax3}	AGT	CAT	GTG	TGC	TAC	TGC	ACA	CAT	AGC	CAA	GGA	TAC	GCG	GGT	AGC	TAT
	S	H	V	C	Y	C	T	H	S	Q	G	Y	A	G	S	Y
$N^{-}6$	AGT	CAT	GTG	TGC	TAC	TGC	···	···	GGA	TAC	GCG	GGT	AGC	TAT		
	S	H	V	C	Y	C	–	–	G	Y	A	G	S	Y		
$N^{-}1$	AGT	CAT	GTG	TGC	TAC	TGC	···	···	···	···	GCG	GGT	GGC	TAT		
	S	H	V	C	Y	C	–	–	–	–	A	G	G	Y		
$N^{-}2$	AGT	···	···	···	···	···	···	CAA	GGA	TAC	GCG	GGT	AGC	TAT		
	S	–	–	–	–	–	–	Q	G	Y	A	G	S	Y		
$N^{-}7$	AGT	CCC	CAA	···	···	···	···	···	CGA	TAC	GCG	GGT	AGC	TAT		
	S	P	Q	–	–	–	–	–	R	Y	A	G	S	Y		

Supplementary Figure 6: Sequences of genomic DNA mutations and resulting amino acid alterations in various NHEJ-induced *Notch* alleles. The two N^{+} Insensitive alleles found cause the following amino-acid substitutions: Ser→Tyr for the N^{+IS1} mutation, and

Ser Gln→Tyr Ala for the composite *N*$^{+IS2}$ mutation. Note that six *de novo* NHEJ induced Abruptex alleles (including *N*^{Ax11}and *N*^{Ax103}) delete one amino acid, the *N*^{Ax26} allele results in the insertion of a single Tyr residue, and the *N*^{Ax3} allele results in the insertion of two amino-acids (Thr-Ser). NHEJ-induced *N*$^{-}$ mutations cause deletions ranging from two to six amino-acids.

Supplementary Figure 7

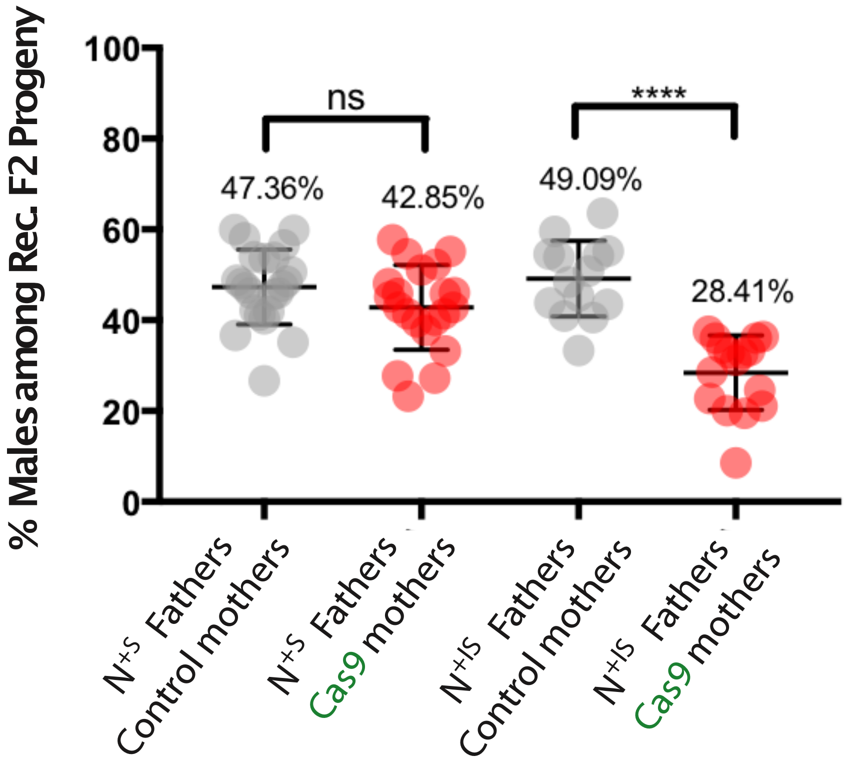

Supplementary Figure 7: Rescuing lethal mosaicism in female F2 progeny skews the fraction of receiver chromosomes recovered in males. Male and female F2 recipients of the receiver chromosome were recovered at approximately equal frequencies when fathers provided a N^{+} sensitive allele (N^{+S}). In contrast, when fathers provided a N^{+} insensitive allele (N^{+IS}), which protects female offspring from lethal mosaicism, a strong skewing against males was observed among F2 progeny inheriting a receiver chromosome. This is additional evidence for lethal mosaicism in F2 females, in which the paternal *N* allele determines lethality (N^{+S}), or survival (N^{+IS}) of the heterozygous female offspring.

Supplementary Figure 8

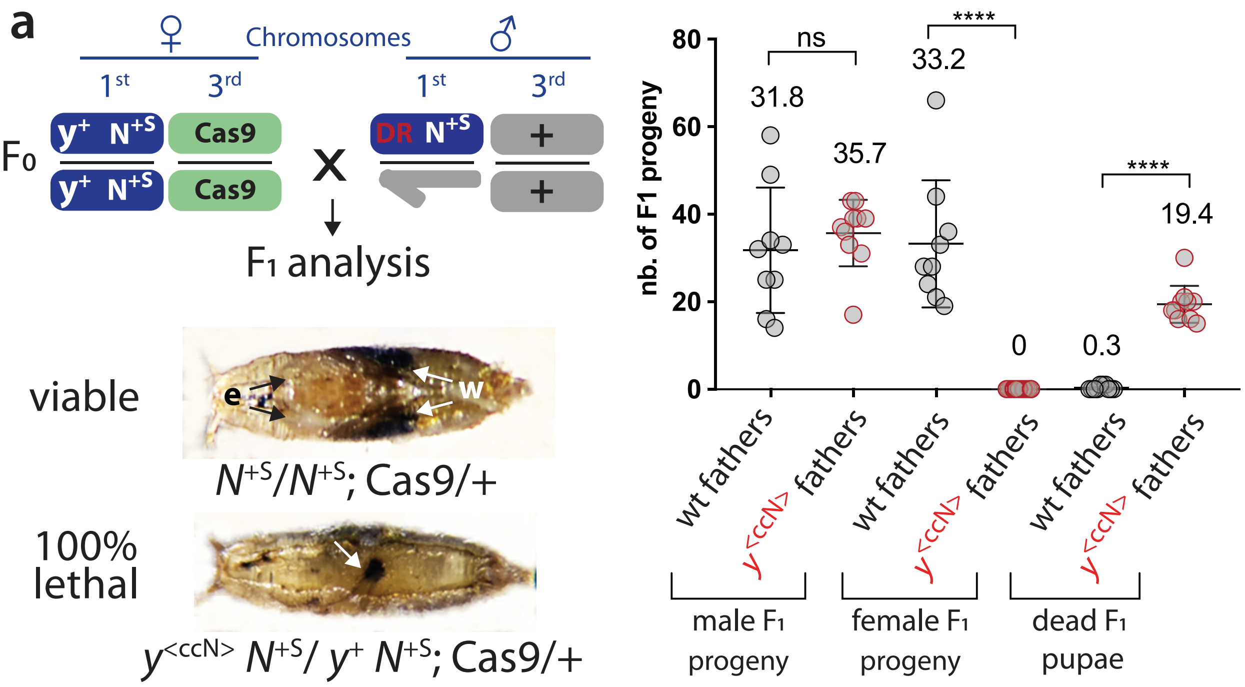

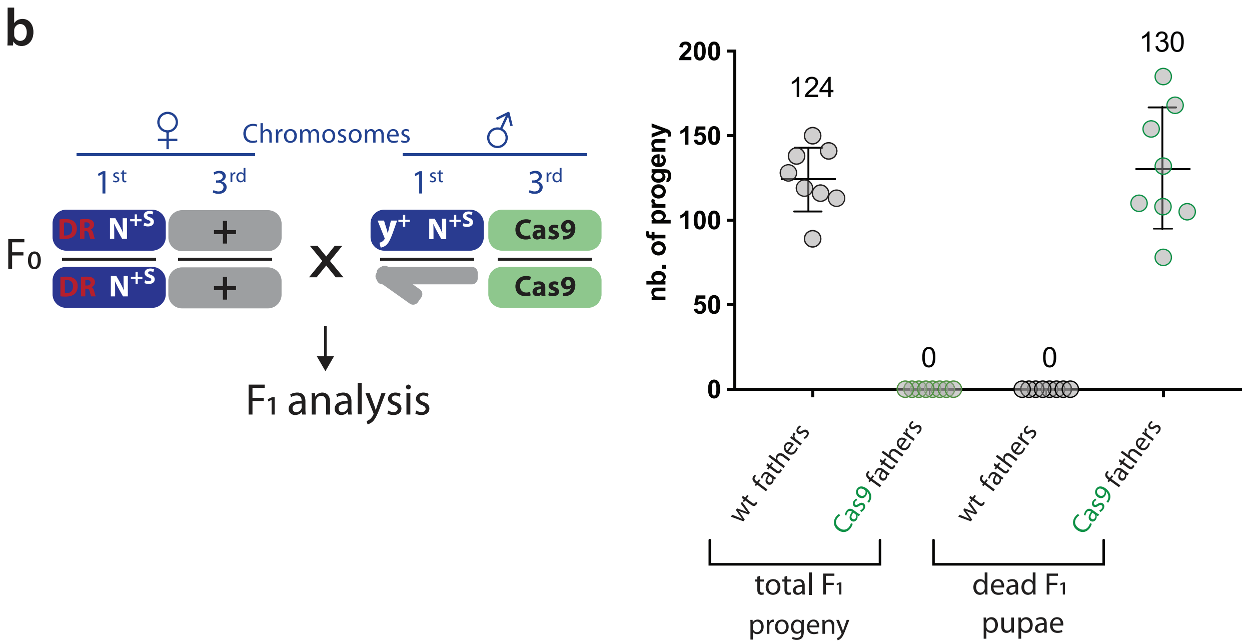

Supplementary Figure 8: 100% lethality induced by the *ccN* CopyCat element in presence of two sensitive *Notch* wild-type alleles and Cas9. **a)** Left panel: males carrying the *ccN* element and a wild-type *Notch* allele were crossed to females carrying two wild-type *Notch* (N^{+S}) alleles and a homozygous *vasa-Cas9* transgene on the third chromosome. Right panel: no viable female progeny were recovered from this cross (circles with red outlines), while male progeny that do not inherit the *ccN* element were recovered in similar numbers as in control crosses (circles with black outlines). Many of the female progeny died at early pupal stages. Lower left panel: a late stage control pupa of the genotype N^{+S}/N^{+S}; Cas9/+ (black arrows labeled "e" indicate the developing eye primordium and white arrows labeled w, point to the wing primordium): Bottom left panel: a dead early stage female pupa of the genotype: $y^{<ccN>}$ N^{+S}/N^{+S}; Cas9/+ (no eye or wing primordia are visible, but necrotic bodies are apparent, white arrow). **b)** Females carrying the *ccN* element and two wild-type *Notch* alleles were crossed to males carrying a wild-type *Notch* allele and also homozygous for the *vasa-Cas9* transgene. No viable progeny were recovered from this cross since they all carried the *ccN* element, a wild-type *Notch* allele, and Cas9.

Supplementary Figure 9

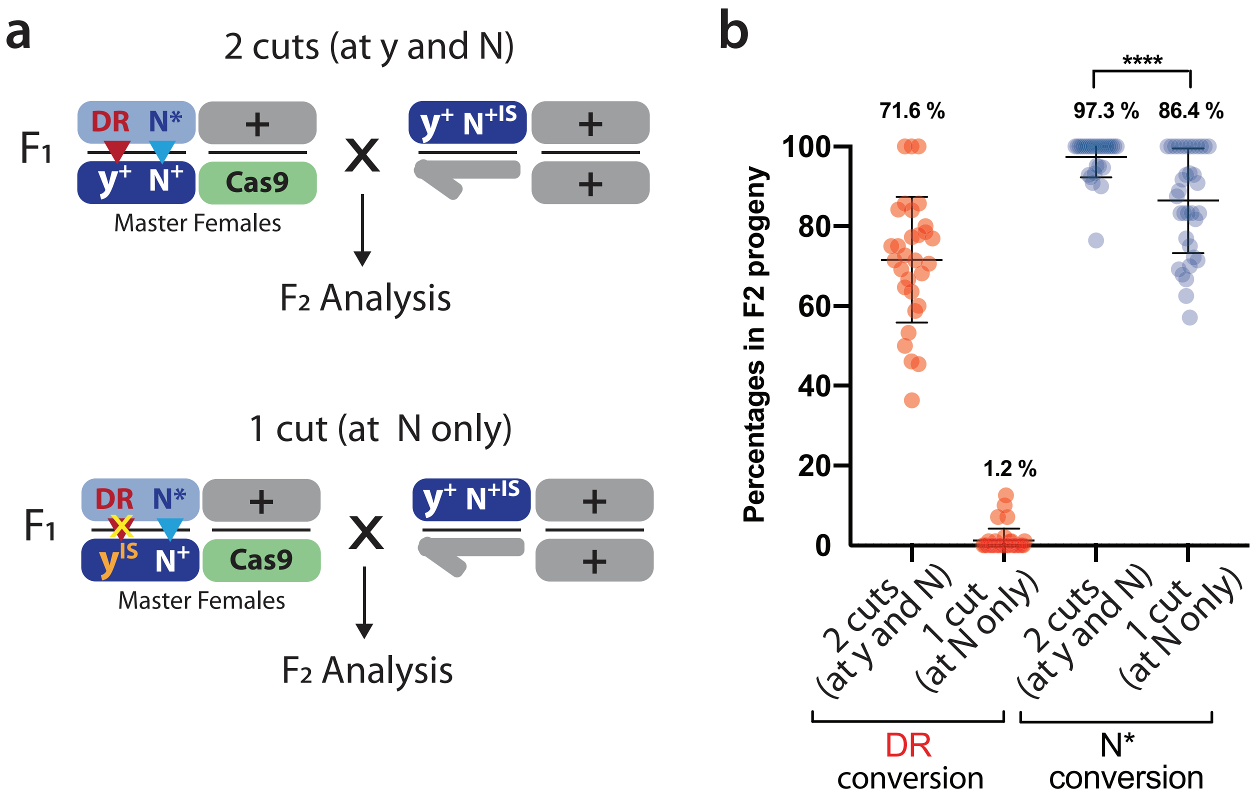

Supplementary Figure 9: Allelic conversion at the *Notch* locus is reduced when copying of the *ccN* element at *yellow* is blocked. **a)** Crossing scheme. Top (double conversion – see results presented in Figure 1F): control copying of both the DsRed (DR) marked $y^{<ccN>}$ and Ax (N*) alleles, which leads to co-drive and high level copying of the Ax allele (97.3% in this experiment - panel b); Bottom (single conversion – see results in panel b): copying of the DsRed marked *ccN* element is blocked by a cleavage insensitive NHEJ allele at the y-gRNA cut site leading to no drive of that element and reduced drive of the Ax allele (panel b). **b)** Conversion percentages for double and

single conversion schemes showing complete inhibition of *ccN* element copying by the *y*- NHEJ allele at the y-gRNA cut site and reduced (86.4%) copying of the Ax allele relative to that in control double conversion crosses (97.3%). Note that the observed levels of conversion for both the *ccN* element and Ax16 allele in control double conversion crosses were higher than in previous experiments, which may reflect the fact that an additional generation was required to generate master females for these crosses, which perhaps reduced the effect of suppressor mutations limiting Cas9 expression/activity. Such suppressors often accumulate in stocks over time.

Supplementary Figure 10

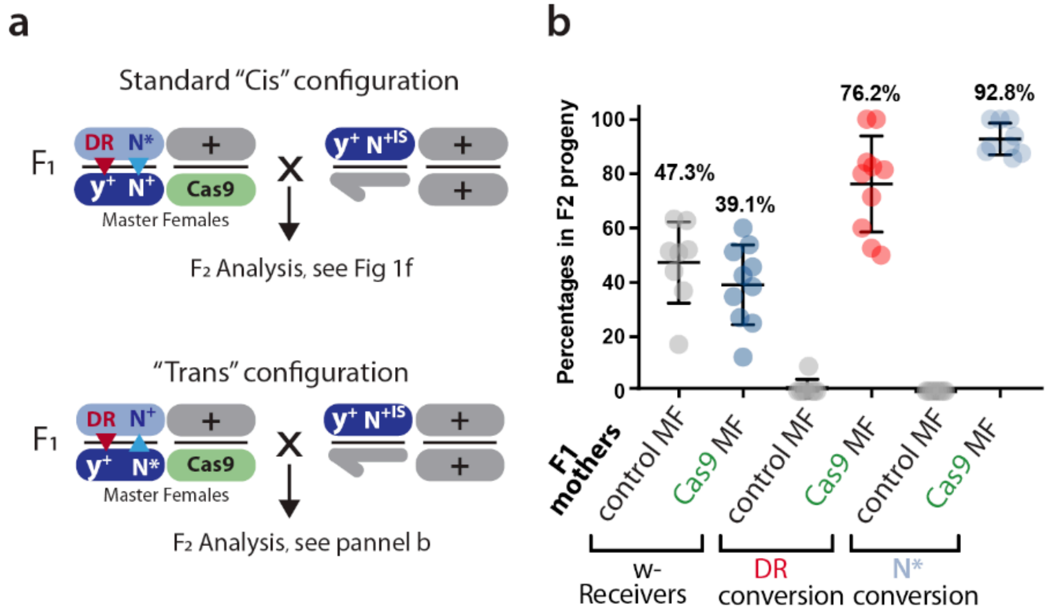

Supplementary Figure 10: Efficient allelic drive is observed when the N^{Ax16} allele is in a trans-configuration relative to the $y^{<ccN>}$ allele. **a)** Crossing schemes depicting the standard cis-conversion drive (top – see results presented in Figure 1F) and trans-configuration drive (panel b) wherein the DsRed marked *ccN* element is linked to the w^{-} and N^{+S} alleles, and the N^{Ax16} allele is on a *y*+ w^{a} chromosome. **b)** Percentages of bi-directional conversion rates (e.g., DsRed w^{a} or Ax w^{-}). Both trans-copying events occur with high efficiency.

Supplementary Figure 11

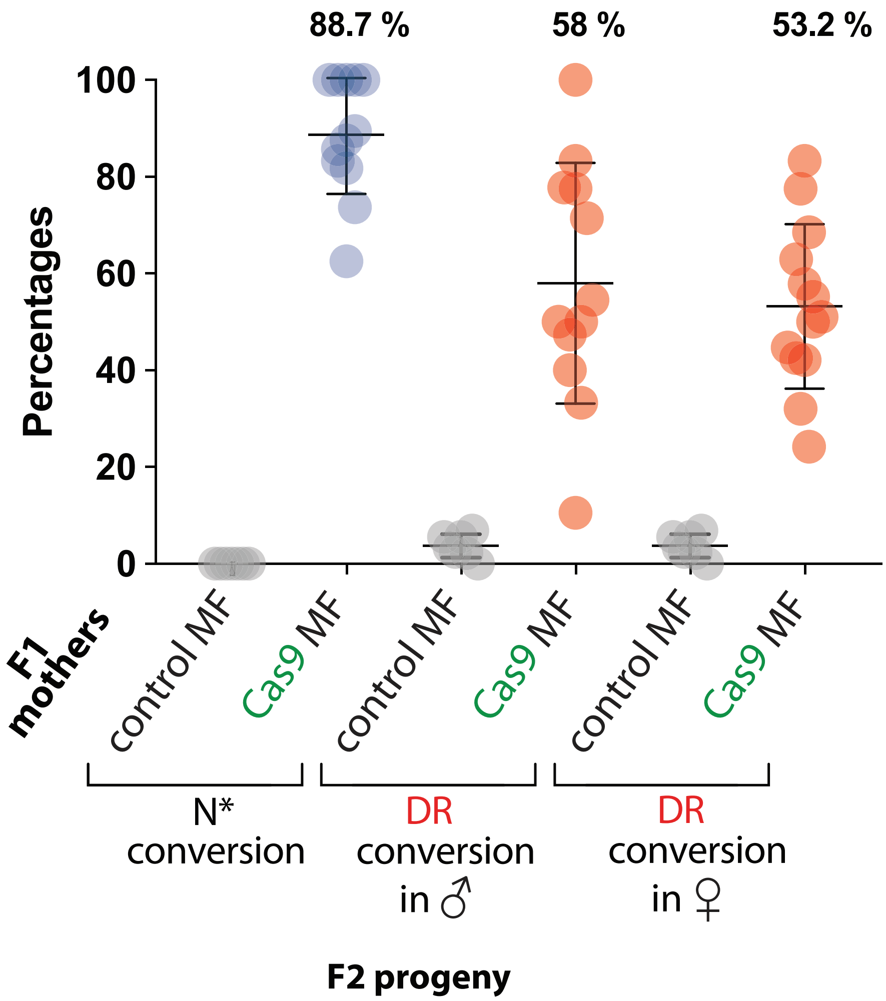

Supplementary Figure 11: Efficient allelic drive of the *de novo* cleavage site N^{Ax103} allele. The *de novo* N^{Ax103} allele (recovered independently 4 times) is an in-frame deletion of 3 nucleotides spanning positions -3 to -5 relative to the PAM sequence that eliminates a serine codon present in the wild-type Notch sequence. Master females carrying the N^{Ax103} allele transmitted the DsRed and Ax phenotype and converted the receiver (w^{-}) target chromosome to Ax at frequencies similar to those observed with the

PAM disrupting N^{Ax16} allele. These results suggest that copy-cutting is also efficient for various alleles that disrupt residues critical for gRNA target site recognition.

Supplementary Figure 12

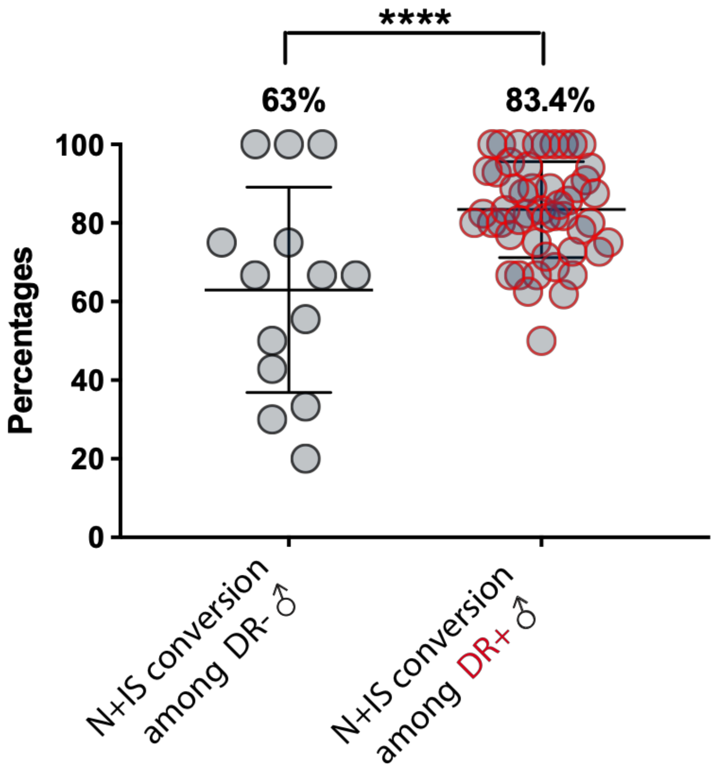

Supplementary Figure 12: Copy-grafting sustains efficient co-drive. The frequency of allelic conversion to the N^{+IS} phenotype among receiver chromosomes (marked with the w^{+} allele) was tabulated among individuals in which the $y^{<ccN>}$ CopyCat allele had also copied or not. As in the case of copy-cutting drive of the N^{Ax16} allele, we observed strong enrichment of N^{+IS} copying events among those that had also copied the $y^{<ccN>}$ allele relative to those that did not.

Supplementary Figure 13

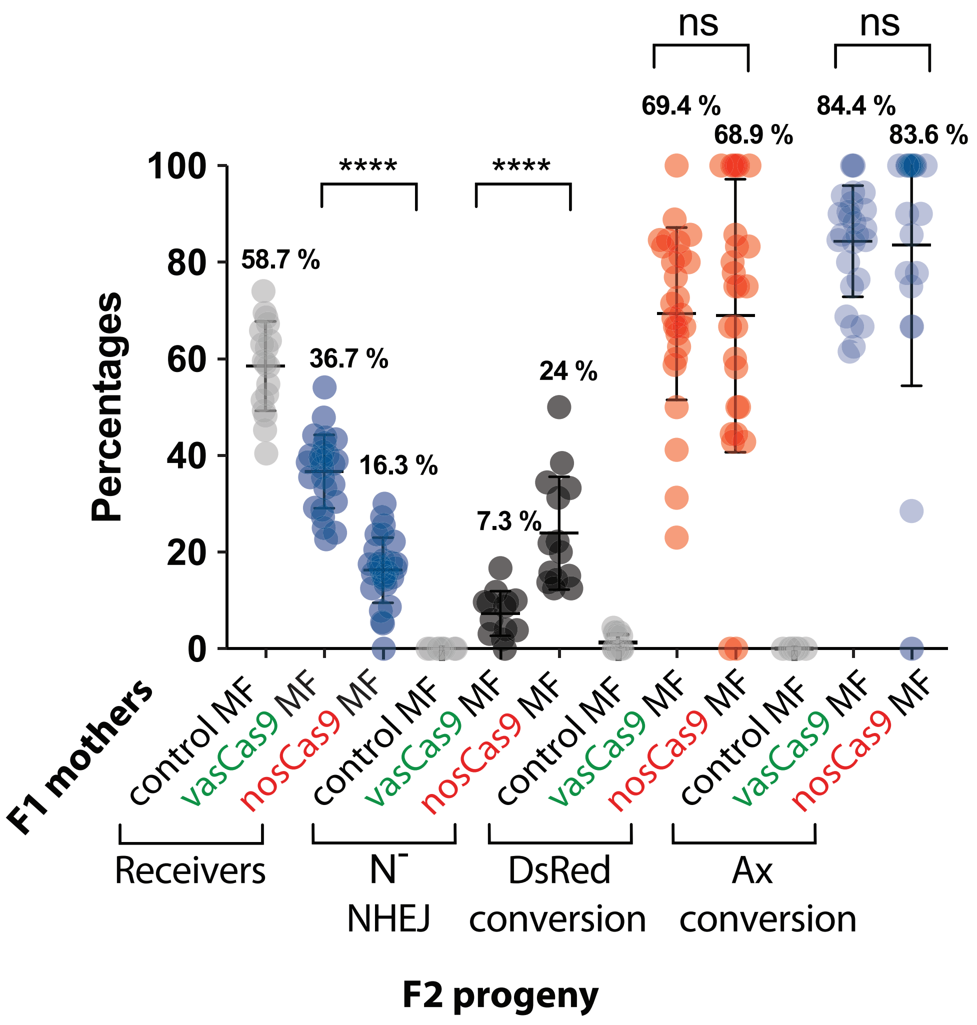

Supplementary Figure 13: The *nanos*-Cas9 source sustains efficient allelic conversion, but also induces more male lethal NHEJ events than observed with the *vasa*-Cas9 source. From left to right: Percentages of F2 progeny inheriting a Receiver chromosome (dark blue dots). Control crosses show a notable bias in favor of the w^-

receiver chromosome allele (~60% transmission) in male progeny, presumably due to a fitness cost associated with the N^{Ax16} allele present on the donor chromosome. Progeny from *nanos*-Cas9 master females show a much stronger reduction in receiver chromosome inheritance (16.3%) in comparison to what is observed with *vasa*-Cas9 (36.7%) master females (MF). This reduction is accompanied by an increase in the frequency of N^{-} NHEJ alleles recovered among F2 Receiver females from *nanos*-Cas9 master females (dark grey dots). In contrast**,** the percent conversion of DsRed and the N^{Ax16} allele among receiver progenies from *nanos*-Cas9 versus *vasa*-Cas9 master females is not significantly different. Consistent with the hypothesis that the *nanos*-Cas9 source generates lethal *Notch* alleles more often than *vasa*-Cas9, a higher frequency of N^{-}/N^{+IS} F2 females were recovered using *nanos*-Cas9 versus *vasa*-Cas9. Cumulatively these data suggest that the modest increase in apparent efficiency of the *nanos*-Cas9 versus *vasa*-Cas9 source in $y^{<ccN>}$ and N^{Ax16} transmission reflects increased rates of generating lethal NHEJ (*Notch*-) alleles rather than an increase in the rate of copying (conversion).

Supplementary Table 1: Complete DNA sequence of ccN CopyCat element.

```
>pVG209_y1-Notch_in_y1-DSRED 2.dna  (8907 bp)
ACCGCCTCTCCCCGCGCGTTGGCCGATTCATTAATGCAGCTGGCACGACAGGTTTCCCGACTGGAAAGCGGGCAGTGAGC
GCAACGCAATTAATGTGAGTTAGCTCACTCATTAGGCACCCCAGGCTTTACACTTTATGCTTCCGGCTCGTATGTTGTGT
GGAATTGTGAGCGGATAACAATTTCACACAGGAAACAGCTATGACCATGATTACGCCAAGCTATTTAGGTGACACTATAG
AATACTCAAGCTATGCATCAAGCTTGGTACCGAGCTCGGATCCACTAGTAACGGCCGCCAGTGTGCTGGAATTCGCCCTT
ctggagactacattgcctgaattggcgggcaaataagtgcgacttggaggaggcggcgaggaagcccagctggtctgagg
tttctgtggcaagacaggacgatattgtttcgtatataacggtggacccattggcaaaacggcttgttttggtattgaaa
cggtatttggcggcccataggcgttattcctcaaatcacacacagtattctcaattaaagtggccaagggagccgtgtaa
attcggaaattagtatctgaataatccaagtcagacagcaagaaaacgggcatcctatcggatagaacccaaacgttttt
gttctcatcaattttcacatcggccggaaaaactaagccaacgtcatcgcgatccacaatgccatgaaattgcggtgagt
acggcattgatgagtgccagcaacccactgcattttgatctattaaattgaacagctcaattccatcatcgctcatcaca
cgtgaagtggtatgggagtttggaccccgttcatctaaggcaacaaagtcatgatagctatcttccgtcctggtttcatc
cctcaaaatcctcgtggatacggcaaattgtcgatgacttgctaacggactaaagtacagggtacgataaccatccgatc
gaatgggcgaaagggacataccaaatataccctcctcgccccattggaagttaataccagcgacattgaaatcgcccctc
aatggatcggggaaaaaatacgaatgtgccgagaatctccaggacttgttcagttcccaggagtaagcaatcaagccgta
tcccaattcatcggcaaaataggcatatgcatcatcgcaattttgcctatatccacggcaatgttagctatgaaagtat
ttggatttgtgtccacgccaggtagctcgtatctccgaattcgcgtatccgtggtcaagtcaaagacatttaccgcatag
gggcacggattagtGGTGGTATTGCCGATGCCCACGGtcgacCGAGCTCGCCCGGGGATCTAATTCAATTAGAGACTAAT
TCAATTAGAGCTAATTCAATTAGGATCCAAGCTTATCGATTTCGAACCCTCGACCGCCGGAGTATAAATAGAGGCGCTTC
GTCTACGGAGCGACAATTCAATTCAAACAAGCAAAGTGAACACGTCGCTAAGCGAAAGCTAAGCAAATAAACAAGCGCAG
CTGAACAAGCTAAACAATCGGCTCGAAGCCGGTCGCCACCatgGCCTCCTCCGAGGACGTCATCAAGGAGTTCATGCGCT
TCAAGGTGCGCATGGAGGGCTCCGTGAACGGCCACGAGTTCGAGATCGAGGGCGAGGGCGAGGGCCGCCCCTACGAGGGC
ACCCAGACCGCCAAGCTGAAGGTGACCAAGGGCGGCCCCCTGCCCTTCGCCTGGGACATCCTGTCCCCCCAGTTCCAGTA
CGGCTCCAAGGTGTACGTGAAGCACCCCGCCGACATCCCCGACTACAAGAAGCTGTCCTTCCCCGAGGGCTTCAAGTGGG
AGCGCGTGATGAACTTCGAGGACGGCGGCGTGGTGACCGTGACCCAGGACTCCTCCCTcCAGGACGGCTCCTTCATCTAC
AAGGTGAAGTTCATCGGCGTGAACTTCCCCTCCGACGGCCCCGTAATGCAGAAGAAGACTATGGGCTGGGAGGCgTCCAC
CGAGCGCCTGTACCCCCGCGACGGCGTGCTGAAGGGCGAGATCCACAAGGCCCTGAAGCTGAAGGACGGCGGCCACTACC
TGGTGGAGTTCAAGTCCATCTACATGGCCAAGAAGCCCGTGCAGCTGCCCGGCTACTACTACGTGGACTCCAAGCTGGAC
ATCACCTCCCACAACGAGGACTACACCATCGTGGAGCAGTACGAGCGCGCCGAGGGCCGCCACCACCTGTTCCTGTAGgg
gccGCGACTCTAGATCATAATCAGCCATACCACATTTGTAGAGGTTTTACTTGCTTTAAAAAACCTCCCACACCTCCCCC
TGAACCTGAAACATAAAATGAATGCAATTGTTGTTGTTAACTTGTTTATTGCAGCTTATAATGGTTACaaataaaGCAAT
AGCATCACAAATTTCACaaataaaGCATTTTTTTCACTGCATTCTAGTTGTGGTTTGTCCAAACTCATCAATGTATCtta
AAGCTTATCGATACGCGTACgctagcACAAAAGCTGGAGCTCCTGCAGGTTGTTGGTTGGCACACCACAAATATACTGTT
GCCGAGCACAATTGTCTAGAATGCATACGCATTAAGCGAACATTAAAAAGATGTGAAAACATAACTATTATGTCTAAATA
AACACACGTCAGATGTATGTACGTCAACGGAAAACCATTGTCTATATATTACAATTACTAAATACATACCAAATTGAATA
CATATTGATGAAAAATAATAAATACTGGCGAAAGCAAAAAAACGAAACATTTTTATTTTATTGAACAACTCTCAGGCTCC
AGGTAGGCAAAAAAGCACCGACTCGGTGCCACTTTTTCAAGTTGATAACGGACTAGCCTTATTTTAACTTGCTATTTCTA
GCTCTAAAACCGGTTCCAGTGTCCAAAACCGACGTTAAATTGAAAATAGGTCTATATATACGAACTGAGTCTGGAAAAAG
AAGTTGAGAATTATAAAAAGTAGTGAGCACTGGCGCCCTCTCTGCTTGGCGAGCTAACCTTTTCGCCTCTTGGCTGAGTA
GGTGGCGTTTCATTCTACTCTGTAAAATTAATGTAGAATTGAAACACTATGGTCAAAAAATACTTAGGGGCATAAGTTAT
AAAACGTATGAAATGAATTTTTATCAACCTGGGCTATTCAAAAATTTTCGAATTATTTTATGTATTTTTTTTAATCGTTT
TTCATTATAGGTTAAAATACACTTTAAAAGGAATTCTTTCCTGTAAAATAAATATAAATAAATATGCTTTATTGACAGAA
AATTTGATGTTTTTGTATTTGAGTAGGAGCAATCACAGGTGAGCAAAAAAGCACCGACTCGGTGCCACTTTTTCAAGTTG
ATAACGGACTAGCCTTATTTTAACTTGCTATTTCTAGCTCTAAAACtgggagcagtagcacacatgcCGAAGTTCACCCG
GATATCTTTCCTATATATACTGTACTCTTGCAGAATATGTACTTGGGAATCATAAGGATGCGAAGGACGTACGCGCTGTG
TCTGTAACTAAGGGGTTGCTTTCGGGTACCTACCATGCCAATGATGGGACTACCGCCGGATAGGAATCCCTTTTGGGTGC
ACACCGTCACCTCCAGTTCCGTCTGCCTGAGTTCGGCAATGGAAATCAGGTACTCAAAGGATGCATCGTAGACGGGGTTG
CAGTTGTCCTTGATCACGCTCGTCTTGCGTTTCGACTCCTTGGTGCGTCCAGGCAACAGATACAGCTTAACATACGGATC
GGGGATATTGCTGGGATCGCGAAGTGGTATCTTCTGGATTTTGTGTATGGTCACGTCTAGTTTTTGACGCTGGGCGCTGT
AGCGGATCGACAACTGCATTCGTCCAAGGCCATGTTCGCCGGGTGAGGAGGTGGAGTCTGGAAAGCGGTGGGTCAGCACA
GAGGTGGCCAGCTCGCTGATGGGTTTTTCGCTGGAGGTGGAGGCCGACATGGCCGGGGAGATTTTCGTAGCCGCCACAGG
ATCTTCTTCACTGATGGGCGATTCGGCGGACAAACGCTTGCTGGATGCCTGAGATTCCTAAAAGCAATGGCAAATTAGAT
TCAATTAAAACAAAAATTGTTTATATAACAAAGTAAAAGAAAAAAAAAAAGAATCACAGATTCTCCCCGACGGGGAATTG
AACCCCGGTCTCCCGCGTGACAGGCGGGGATACTAACCACTATACTATCGAGGACGTTGAAAATgacgtcttccagtgtc
caaaacccacagccgaccacactcatccactttaatgcggtaggcagtggtaatactgttggcgcaatctccagctgtat
ttgagcgccaatctggatacggaattagctccggtgaacccgtcaaactgcggtccatgtttatataggtcagagtggcc
ggaatccctaaaaaataaaataggagaattagcagggccaaactattattgaggcactaaataaagtttgttttgaatat
ttaattaactggaatttctgcctaaattccttctttggattattatactaagcaatgttgcaagttatcgctgtctgttt
tctggataatatgtaaatgcatacagatgtaaaggtaaattagaatcgcttatgtttggcatttaattatttatatacct
```

atttgtcattattttggacacaaggaatcataagcaatggtaactgccgtgctaatggctctttttctgcttgtacatc
tactggaagtttatgttaaaagatccccggctatatgtacttcagtcataattggcactattgcaagttgcgtgcaattt
gcgatgtcattagccgtctgaagcaattgcgtctgacatcattatcaatatgtataccatatatcgctgttaaatgtata
tgtggccattggcagtatttcgaccgatttccgctccagttcttttccttctttatttcttcgttaattgaatttaaaga
catttattgtttctgggaacttttaatgtttttatagcaggcgagtgagactgcaacgaccagaaaatatagcttttatc
gatttaccgacccatcttcttaaaaatcaaaccaggcgtttttgctttacaaagtaaccggggggttgcgataaatataca
tttgagcataatgcacctttcaatctttatttaattcataacttttaaaataacaatctaattcagtcaaatataaagta
tttgaacaaatttatatttgacatgtgctctttcagtcctaaaacctcgcaacAAGGGCGAATTCTGCAGATATCCATC
ACACTGGCGGCCGCTCGAGCATGCATCTAGAGGGCCCAATTCGCCCTATAGTGAGTCGTATTACAATTCACTGGCCGTCG
TTTTACAACGTCGTGACTGGGAAAACCCTGGCGTTACCCAACTTAATCGCCTTGCAGCACATCCCCCTTTCGCCAGCTGG
CGTAATAGCGAAGAGGCCCGCACCGATCGCCCTTCCCAACAGTTGCGCAGCCTGAATGGCGAATGGACGCGCCCTGTAGC
GGCGCATTAAGCGCGGCGGGTGTGGTGGTTACGCGCAGCGTGACCGCTACACTTGCCAGCGCCCTAGCGCCCGCTCCTTT
CGCTTTCTTCCCTTCCTTTCTCGCCACGTTCGCCGGCTTTCCCCGTCAAGCTCTAAATCGGGGGCTCCCTTTAGGGTTCC
GATTTAGTGCTTTACGGCACCTCGACCCCAAAAAACTTGATTAGGGTGATGGTTCACGTAGTGGGCCATCGCCCTGATAG
ACGGTTTTTCGCCCTTTGACGTTGGAGTCCACGTTCTTTAATAGTGGACTCTTGTTCCAAACTGGAACAACACTCAACCC
TATCTCGGTCTATTCTTTTGATTTATAAGGGATTTTGCCGATTTCGGCCTATTGGTTAAAAAATGAGCTGATTTAACAAA
AATTTAACGCGAATTTTAACAAAATTCAGGGCGCAAGGGCTGCTAAAGGAAGCGGAACACGTAGAAAGCCAGTCCGCAGA
AACGGTGCTGACCCCGGATGAATGTCAGCTACTGGGCTATCTGGACAAGGGAAAACGCAAGCGCAAAGAGAAAGCAGGTA
GCTTGCAGTGGGCTTACATGGCGATAGCTAGACTGGGCGGTTTTATGGACAGCAAGCGAACCGGAATTGCCAGCTGGGGC
GCCCTCTGGTAAGGTTGGGAAGCCCTGCAAAGTAAACTGGATGGCTTTCTTGCCGCCAAGGATCTGATGGCGCAGGGGAT
CAAGATCTGATCAAGAGACAGGATGAGGATCGTTTCGCATGATTGAACAAGATGGATTGCACGCAGGTTCTCCGGCCGCT
TGGGTGGAGAGGCTATTCGGCTATGACTGGGCACAACAGACAATCGGCTGCTCTGATGCCGCCGTGTTCCGGCTGTCAGC
GCAGGGGCGCCCGGTTCTTTTTGTCAAGACCGACCTGTCCGGTGCCCTGAATGAACTGCAGGACGAGGCAGCGCGGCTAT
CGTGGCTGGCCACGACGGGCGTTCCTTGCGCAGCTGTGCTCGACGTTGTCACTGAAGCGGGAAGGGACTGGCTGCTATTG
GGCGAAGTGCCGGGGCAGGATCTCCTGTCATCCCACCTTGCTCCTGCCGAGAAAGTATCCATCATGGCTGATGCAATGCG
GCGGCTGCATACGCTTGATCCGGCTACCTGCCCATTCGACCACCAAGCGAAACATCGCATCGAGCGAGCACGTACTCGGA
TGGAAGCCGGTCTTGTCGATCAGGATGATCTGGACGAAGAGCATCAGGGGCTCGCGCCAGCCGAACTGTTCGCCAGGCTC
AAGGCGCGCATGCCCGACGGCGAGGATCTCGTCGTGACCCATGGCGATGCCTGCTTGCCGAATATCATGGTGGAAAATGG
CCGCTTTTCTGGATTCATCGACTGTGGCCGGCTGGGTGTGGCGGACCGCTATCAGGACATAGCGTTGGCTACCCGTGATA
TTGCTGAAGAGCTTGGCGGCGAATGGGCTGACCGCTTCCTCGTGCTTTACGGTATCGCCGCTCCCGATTCGCAGCGCATC
GCCTTCTATCGCCTTCTTGACGAGTTCTTCTGAATTGAAAAAGGAAGAGTATGAGTATTCAACATTTCCGTGTCGCCCTT
ATTCCCTTTTTTGCGGCATTTTGCCTTCCTGTTTTTGCTCACCCAGAAACGCTGGTGAAAGTAAAAGATGCTGAAGATCA
GTTGGGTGCACGAGTGGGTTACATCGAACTGGATCTCAACAGCGGTAAGATCCTTGAGAGTTTTCGCCCCGAAGAACGTT
TTCCAATGATGAGCACTTTTAAAGTTCTGCTATGTGGCGCGGTATTATCCCGTATTGACGCCGGGCAAGAGCAACTCGGT
CGCCGCATACACTATTCTCAGAATGACTTGGTTGAGTACTCACCAGTCACAGAAAAGCATCTTACGGATGGCATGACAGT
AAGAGAATTATGCAGTGCTGCCATAACCATGAGTGATAACACTGCGGCCAACTTACTTCTGACAACGATCGGAGGACCGA
AGGAGCTAACCGCTTTTTTGCACAACATGGGGGATCATGTAACTCGCCTTGATCGTTGGGAACCGGAGCTGAATGAAGCC
ATACCAAACGACGAGCGTGACACCACGATGCCTGTAGCAATGGCAACAACGTTGCGCAAACTATTAACTGGCGAACTACT
TACTCTAGCTTCCCGGCAACAATTAATAGACTGGATGGAGGCGGATAAAGTTGCAGGACCACTTCTGCGCTCGGCCCTTC
CGGCTGGCTGGTTTATTGCTGATAAATCTGGAGCCGGTGAGCGTGGGTCTCGCGGTATCATTGCAGCACTGGGGCCAGAT
GGTAAGCCCTCCCGTATCGTAGTTATCTACACGACGGGGAGTCAGGCAACTATGGATGAACGAAATAGACAGATCGCTGA
GATAGGTGCCTCACTGATTAAGCATTGGTAACTGTCAGACCAAGTTTACTCATATATACTTTAGATTGATTTAAAACTTC
ATTTTTAATTTAAAAGGATCTAGGTGAAGATCCTTTTTGATAATCTCATGACCAAAATCCCTTAACGTGAGTTTTCGTTC
CACTGAGCGTCAGACCCCGTAGAAAAGATCAAAGGATCTTCTTGAGATCCTTTTTTTCTGCGCGTAATCTGCTGCTTGCA
AACAAAAAAACCACCGCTACCAGCGGTGGTTTGTTTGCCGGATCAAGAGCTACCAACTCTTTTTCCGAAGGTAACTGGCT
TCAGCAGAGCGCAGATACCAAATACTGTTCTTCTAGTGTAGCCGTAGTTAGGCCACCACTTCAAGAACTCTGTAGCACCG
CCTACATACCTCGCTCTGCTAATCCTGTTACCAGTGGCTGCTGCCAGTGGCGATAAGTCGTGTCTTACCGGGTTGGACTC
AAGACGATAGTTACCGGATAAGGCGCAGCGGTCGGGCTGAACGGGGGGTTCGTGCACACAGCCCAGCTTGGAGCGAACGA
CCTACACCGAACTGAGATACCTACAGCGTGAGCTATGAGAAAGCGCCACGCTTCCCGAAGGGAGAAAGGCGGACAGGTAT
CCGGTAAGCGGCAGGGTCGGAACAGGAGAGCGCACGAGGGAGCTTCCAGGGGGAAACGCCTGGTATCTTTATAGTCCTGT
CGGGTTTCGCCACCTCTGACTTGAGCGTCGATTTTTGTGATGCTCGTCAGGGGGGCGGAGCCTATGGAAAAACGCCAGCA
ACGCGGCCTTTTTACGGTTCCTGGCCTTTTGCTGGCCTTTTGCTCACATGTTCTTTCCTGCGTTATCCCCTGATTCTGTG
GATAACCGTATTACCGCCTTTGAGTGAGCTGATACCGCTCGCCGCAGCCGAACGACCGAGCGCAGCGAGTCAGTGAGCGA
GGAAGCGGAAGAGCGCCCAATACGCAA

Supplementary Table 2: Phenotypic and molecular analysis of 104 isogenic F2 lines.

line #	DsRed	Donor or Receiver	N+/Ax16 phenotype	y+ or y-	NAx region Sequence	Category	Comments
**A1**	+	D	Ax	y-		DON	
**A2**	+	D	Ax	y-		DON	
**A4**	+	R	Ax	y-	Ax16	DCR	
**A5**	+	R	Ax	y-	Ax16	DCR	
**A6**	+	R	Ax	y-	Ax16	DCR	
**A7**	+	D	Ax	y-		DON	
**A8**	+	D	Ax	y-		DON	
**A9**	+	D	Ax	y-		DON	
**A10**	+	D	Ax	y-		DON	
**A11**	-	R	Ax	y-	3nt deletion at cut site	ACR	new Ax allele
**A12**	+	R	Ax	y-	Ax16	DCR	
**A13**	+	R	N+	y-	N+	RCR	
**A14**	+	D	Ax	y-		DON	
**A15**	+	D	Ax	y-		DON	
**C25**	+	R	Ax	y-	Ax16	DCR	
**C26**	+	R	Ax	y-	Ax16	DCR	
**C27**	+	R	Ax	y-	Ax16	DCR	
**C28**	-	R	N+	y-	N+	INR	
**C29**	+	D	Ax	y-		DON	
**D31**	-	R	Ax	y-	Ax16	ACR	
**D33**	+	R	Ax	y-	Ax16	DCR	
**D34**	+	D	Ax	y-		DON	
**D35**	+	D	Ax	y-		DON	
**D36**	-	R	N+	y-	N+	INR	
**D37**	-	R	N+	y-	N+	INR	
**D39**	+	D	Ax	y-		DON	
**E41**	+	D	Ax	y-		DON	
**E42**	+	R	Ax	y-	Ax16	DCR	
**E43**	+	D	Ax	y-		DON	
**E44**	-	D	Ax	y-		DON	recombination
**E45**	-	R	Ax	y-	Ax16	ACR	
**E47**	+	R	Ax	y-	Ax16	DCR	
**E48**	-	R	Ax	y-	Ax16	ACR	
**E49**	+	R	Ax	y-	Ax16	DCR	
**F50**	+	D	Ax	y-		DON	
**F51**	-	R	Ax	y-	Ax16	ACR	
**G52**	-	R	N+	y-	C to A sub. at position -1	INR	cut-resistant N+ allele
**G53**	-	R	N+	y-	N+	INR	
**G54**	-	R	Ax	y-	Ax16	ACR	
**H55**	-	R	N+	y-	N+	INR	
**H56**	+	D	Ax	y-		DON	

**H57**	+	D	Ax	y-		DON	
**H58**	+	D	Ax	y-		DON	
**H59**	-	R	Ax	y-	Ax16	ACR	
**I60**	-	R	Ax	y-	Ax16	ACR	
**I61**	+	D	Ax	-		DON	
**I62**	-	R	N+	y-	N+	INR	
**I63**	+	R	Ax	y-	Ax16	DCR	
**I64**	+	D	Ax	y-		DON	
**I65**	+	D	Ax	y-		DON	
**I66**	+	D	Ax	y-		DON	
**I67**	+	D	Ax	y-		DON	
**I68**	+	D	Ax	y-		DON	
**K69**	+	D	Ax	y-		DON	
**K70**	-	R	N+	y-	N+	INR	
**K71**	+	D	Ax	y-		DON	
**K72**	+	D	Ax	y-		DON	
**K73**	+	D	Ax	y-		DON	
**K74**	+	D	Ax	y-		DON	
**K76**	+	R	Ax	y-	Ax16	DCR	
**K77**	-	R	Ax	y-	Ax16	ACR	
**K78**	+	D	Ax	y-		DON	
**K79**	-	R	Ax	y-	Ax16	ACR	
**K80**	-	D	Ax	y-		DON	recombination
**K81**	+	D	Ax	y-		DON	
**K82**	-	R	Ax	y-	Ax16	ACR	
**K83**	-	R	Ax	y-	Ax16	ACR	
**K84**	+	D	Ax	y-		DON	
**K85**	+	D	Ax	y-		DON	
**K87**	+	D	Ax	y-		DON	
**K88**	+	R	Ax	y-	Ax16	DCR	
**K89**	+	D	Ax	y-		DON	
**K90**	+	D	Ax	y-		DON	
**K91**	+	D	Ax	y-		DON	
**L92**	+	D	Ax	y-		DON	
**L93**	+	R	Ax	y-	Ax16	DCR	
**L94**	+	R	Ax	y-	Ax16	DCR	
**L95**	+	D	Ax	y-		DON	
**L96**	+	D	Ax	y-		DON	
**L97**	+	D	Ax	y-		DON	
**L98**	+	D	Ax	y-		DON	
**L99**	+	R	Ax	y-	Ax16	DCR	
**M100**	-	R	Ax	y-	Ax16	ACR	
**M101**	-	R	N+	y-	N+	IR	
**M102**	+	R	Ax	y-	Ax16	DCR	
**M103**	+	R	Ax	y-	3 nt deletion at cut site	DCR	new Ax allele

**M104**	-	R	N+	y-	N+	IR	
**M105**	+	D	Ax	y-		DON	
**M106**	+	D	Ax	y-		DON	
**M107**	+	D	Ax	y-		DON	
**M108**	+	D	Ax	y-		DON	
**M109**	-	R	Ax	y-	Ax16	ACR	
**M110**	+	R	Ax	y-	Ax16	DCR	
**M111**	+	R	Ax	y-	Ax16	DCR	
**M112**	+	R	Ax	y-	Ax16	DCR	
**M113**	+	D	Ax	y-		DON	
**M114**	+	D	Ax	y-		DON	
**M115**	-	R	N+	y-	N+	INR	
**M117**	+	D	Ax	y-		DON	
**M118**	+	D	Ax	y-		DON	
**M119**	-	R	N+	y-	N+	INR	
**M120**	+	R	Ax	y-	Ax16	DCR	
**M121**	+	D	Ax	y-		DON	
**M122**	+	D	Ax	y-		DON	

Supplementary Table 2: Phenotypic and molecular analysis of 104 isogenic F2 female lines. **Column1**: line number. **Column 2:** Presence (+) or absence (-) of the DsRed-marked ccN CopyCat element. **Column 3:** origin of the founder chromosome (Donor : D, or Receiver : R), as determined by sequencing a polymorphic site located 6.5 Kb upstream of the Ax16 mutation (Supplementary Fig. 4a). Receiver lines are highlighted in green. **Column 4:** Notch wing phenotype, Abruptex (Ax) or wild-type (N+). **Column 5:** Yellow allele (only y- alleles were recovered). **Column 6:** Sequence analysis of the Ax region from Receiver chromosomes, revealing a strict correspondence between the phenotypic and molecular analysis. In addition, two Ax alleles (lines 11 and 103) were created by non-homologous end joining rather than allelic conversion. **Column 7:** Chromosome category. DON: Donor. DCR: Double converted receiver. INR: Intact receiver. ACR: Ax only converted receiver. RCR: DsRed only converted receiver. **Column 8:** Comments. For lines 44 and 80, recombination separated Ax form the DsRed ccN CopyCat element. For lines 11 and 103, a new Ax alleles was created.

Supplementary Table 3: Summary of phenotypic analysis of 104 isogenic lines

Categories	Abbreviation	#Lines	Percentage
Donor	DON	54	51.92%
Double Conversion	DCR	23	22.11%
Intact receiver	INR	12	11.53%
Ax only Conversion	ACR	14	13.46%
DsRed only Conversion	RCR	1	0.96%
Total		104	
Categories	Includes	#Lines	Percentage
DsRed-	ACR+INR	28	27%
DsRed+	DON+DCR+RCR	76	75%
N+	INR+RCR	13	12.50%
NAx	DON+ACR+DCR	91	87.50%
DsRed Conversion	DCR+RCR	24	48%
NAx Conversion	DCR+ACR	37	74%

Supplementary Table 3: Summary of the different categories of molecular events determined on receiver versus donor chromosomes for the 104 isogenic lines and accompanying abbreviation key for Supplementary Table 2.

Supplementary References

1 Gantz, V. M. & Bier, E. The dawn of active genetics. *Bioessays* **38**, 50-63, doi:10.1002/bies.201500102 (2016).

2 Xu, X. S., Gantz, V. M., Siomava, N. & Bier, E. CRISPR/Cas9 and active genetics-based trans-species replacement of the endogenous Drosophila kni-L2 CRM reveals unexpected complexity. *Elife* **6**, doi:10.7554/eLife.30281 (2017).
